# A transformer-based multi-modal fusion framework for laryngeal lesion classification using contact endoscopy

**DOI:** 10.3389/fsurg.2026.1840300

**Published:** 2026-09-03

**Authors:** Yunjing Duan, Wei Li

**Affiliations:** Department of Otolaryngology – Head and Neck Surgery, Shijiazhuang People's Hospital, Shijiazhuang, Hebei, China

**Keywords:** computer-aided diagnosis, contact endoscopy, deep learning, feature selection, laryngeal cancer, multi-modal fusion, narrow band imaging, radiomics

## Abstract

**Purpose:**

This study introduces a novel multi-modal transformer-based framework that integrates clinical data, radiomic features, and deep learning representations for automated classification of laryngeal lesions from contact endoscopy images.

**Methods:**

We retrospectively enrolled 300 patients with laryngeal lesions from three independent medical centers (Centers A, B, and C), acquiring 7,847 contact endoscopy images with narrow band imaging. Clinical variables (18 features), radiomic features extracted using PyRadiomics (156 features), and deep learning representations from three state-of-the-art architectures, ConvNeXt V2, Swin Transformer V2, and EfficientNet V2 (3,328 combined features), were systematically integrated through a six-layer transformer encoder with multi-head cross-attention mechanisms. We evaluated three feature selection strategies (LASSO, mutual information, ReliefF) and three advanced classification architectures (Vision Transformer, Attention-MLP, Graph Neural Network). The framework was developed using data from Centers A and B (*n* = 230 patients) with rigorous five-fold cross-validation, and validated on an independent external test set from Center C (*n* = 70 patients).

**Results:**

The optimal configuration (LASSO feature selection with Vision Transformer classifier) achieved accuracy of 96.1 ± 1.0% (AUC-ROC: 0.992 ± 0.007) on training, 93.8 ± 1.4% (AUC-ROC: 0.981 ± 0.012) on internal validation, and 91.4 ± 2.1% (AUC-ROC: 0.967 ± 0.019) on external testing, with sensitivity of 90.0 ± 2.8% and specificity of 92.5 ± 2.5% on the external cohort. The multi-modal fusion approach significantly outperformed all single-modality

**Methods:**

clinical features alone (71.3% validation accuracy), radiomic features alone (84.2%), and the best individual deep learning model (87.6%), with improvements of 22.5, 9.6, and 6.2 percentage points respectively (all *p* < 0.001. Attention mechanism analysis revealed that the model dynamically weighted modality contributions, allocating 47.0% attention to deep features, 34.7% to radiomic features, and 18.3% attention to clinical features for correctly classified malignant cases.

**Conclusion:**

This study presents the first comprehensive framework for laryngeal lesion classification that systematically integrates clinical data, radiomics, and deep learning through transformer-based fusion.

## 1 Introduction

Laryngeal cancer represents one of the most prevalent malignancies of the head and neck region, with squamous cell carcinoma accounting for more than 95% of cases (1). Early detection and accurate diagnosis are paramount for improving patient prognosis, preserving laryngeal function, and maintaining quality of life (2). Conventional diagnostic approaches rely primarily on white light endoscopy followed by histopathological examination of biopsy specimens (3). However, these methods are limited by subjective interpretation, inter-observer variability, and invasive tissue sampling (4). Contact endoscopy combined with narrow band imaging has emerged as a promising technique for enhanced visualization of subepithelial vascular patterns within vocal folds, providing real-time, magnified views of microvascular architecture that serve as critical indicators for distinguishing benign from malignant lesions (5). Abnormal vascular patterns, including irregular vessel morphology, increased vessel density, and tortuous configurations, are strongly associated with malignant transformation (6). Nevertheless, interpretation of contact endoscopy images remains heavily dependent on clinician expertise, leading to diagnostic inconsistency, particularly in assessing subtle or early-stage lesions (7). This challenge has motivated substantial research efforts toward developing automated, objective, and reproducible computer-aided diagnostic systems (8).

The advent of artificial intelligence has catalyzed transformative advances in medical image analysis, with deep learning approaches demonstrating remarkable success in automated lesion detection and classification across diverse clinical domains (9). In laryngeal cancer diagnosis, convolutional neural networks have achieved diagnostic accuracies comparable to or exceeding those of experienced otolaryngologists (10). However, existing approaches predominantly rely on single-modality analysis, utilizing either handcrafted radiomic features or learned deep representations (11). Radiomic analysis provides quantitative, reproducible measurements of image characteristics such as texture and vascular patterns, offering interpretable features aligned with established clinical knowledge (12). Conversely, deep learning models excel at discovering hierarchical feature representations that capture subtle patterns imperceptible to conventional methods (13). While both paradigms demonstrate individual merit, their complementary nature suggests that integration could yield superior diagnostic performance (3, 14). Furthermore, incorporating clinical data, including patient demographics, tumor staging, and risk factors, has been shown to enhance predictive models in oncological applications (15). However, no comprehensive framework has systematically integrated clinical variables, radiomic features, and deep learning representations within a unified architecture for laryngeal lesion assessment from contact endoscopy images (16).

Multi-modal fusion techniques have gained considerable attention, with transformer-based architectures emerging as particularly effective mechanisms for learning complex inter-modal relationships through self-attention and cross-attention (17). Unlike traditional concatenation-based strategies, transformers can learn contextual dependencies across modalities, adaptively weighting feature contributions based on diagnostic relevance (18). Despite these advances, transformer-based multi-modal fusion for laryngeal cancer diagnosis remains unexplored. Additionally, feature selection constitutes a critical yet often overlooked component in multi-modal systems. High-dimensional feature spaces inevitably contain redundant or noisy features that compromise model generalization and interpretability. While various algorithms exist, including LASSO regularization, mutual information, and ReliefF, their comparative effectiveness in multi-modal laryngeal lesion classification has not been systematically evaluated (10). Furthermore, the choice of classification architecture following feature fusion warrants careful consideration, as advanced deep classifiers may offer advantages over conventional fully connected layers (19).

Several fundamental challenges must be addressed to develop clinically viable automated diagnostic systems. First, the heterogeneity of data modalities poses significant integration challenges, as clinical variables, radiomic features, and deep representations exhibit vastly different statistical properties and dimensionalities. Second, limited availability of large-scale, multi-center datasets constrains model development, necessitating approaches that achieve strong performance with modest sample sizes while maintaining generalizability across institutions. Third, the black-box nature of deep learning raises concerns regarding clinical interpretability, particularly where understanding prediction rationale is essential for clinician acceptance and regulatory approval (20).

This study introduces a novel multi-modal transformer-based framework integrating clinical data, radiomic features, and deep learning representations for automated laryngeal lesion classification from contact endoscopy images. Our approach employs state-of-the-art architectures including ConvNeXt V2, Swin Transformer V2, and EfficientNet V2 to extract hierarchical feature representations, while deriving quantitative radiomic signatures using PyRadiomics to characterize vascular patterns and textural properties. Patient-specific clinical variables are systematically incorporated to provide complementary contextual information. These three modalities are integrated through a transformer fusion module leveraging multi-head cross-attention mechanisms to learn adaptive fusion weights. We conduct comprehensive comparative analysis of three feature selection strategies, LASSO, mutual information, and ReliefF, evaluating their impacts on classification performance. Following feature selection, we investigate three advanced deep classifiers: a vision transformer, an attention-enhanced multilayer perceptron, and a graph neural network. The framework is validated using a multi-center dataset from three independent institutions, with rigorous five-fold cross-validation for internal validation and an external test set for assessing generalizability.

## 2 Methods

### 2.1 Dataset and patient selection

#### 2.1.1 Inclusion and exclusion criteria

Patients aged 18 years or older presenting with suspected laryngeal lesions who underwent contact endoscopy with narrow band imaging examination were retrospectively enrolled from three independent medical centers between January 2022 and December 2,024. Inclusion criteria required histopathologically confirmed diagnosis (benign or malignant), availability of high-quality contact endoscopy images showing clear vascular patterns, and complete clinical records including demographic information and tumor characteristics.

**Figure 1:**
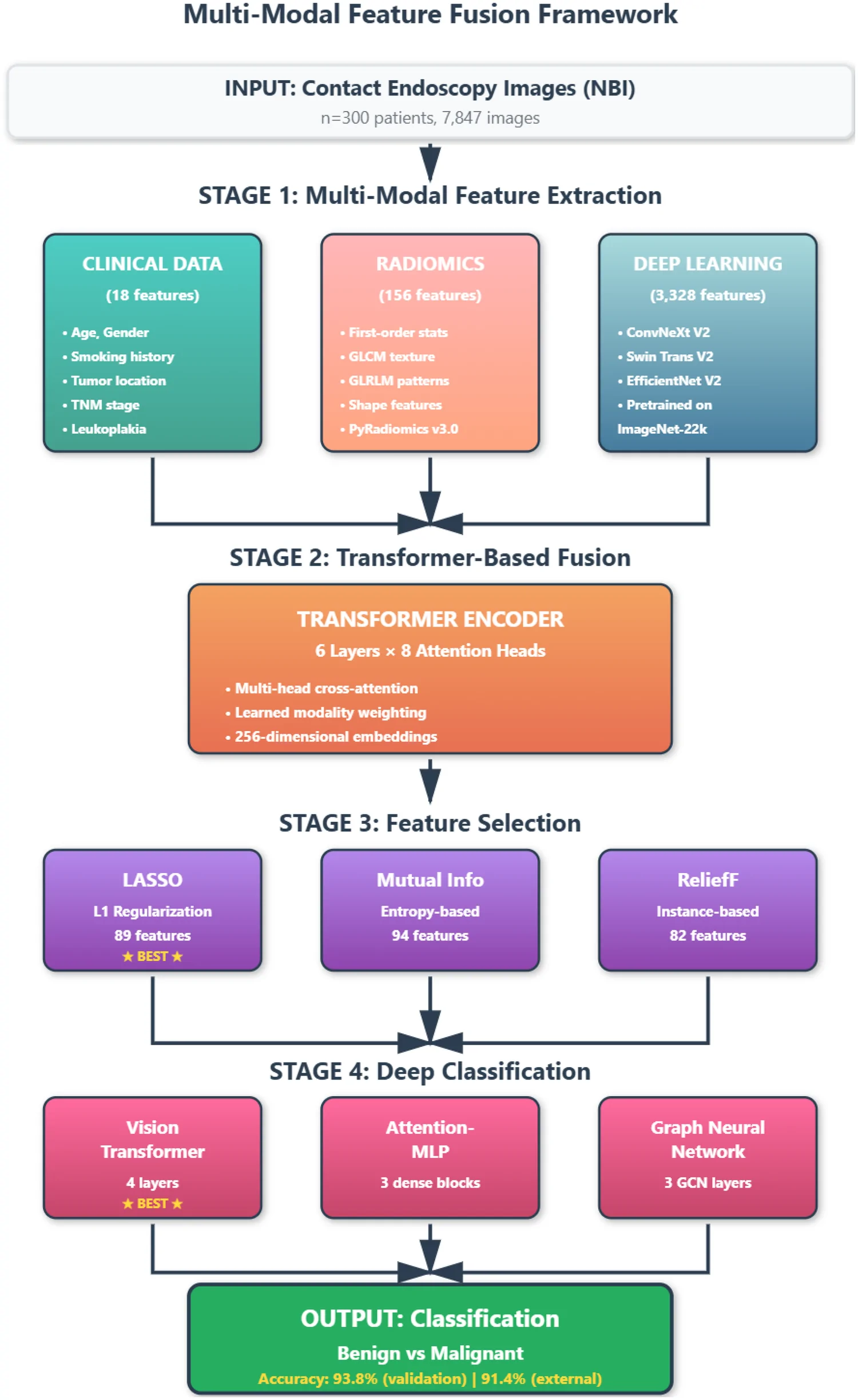
Overall framework architecture.

Exclusion criteria comprised patients with prior radiation therapy or chemotherapy, insufficient image quality due to motion artifacts or bleeding, incomplete clinical data, lesions smaller than 3 mm that precluded adequate vascular pattern visualization, and patients who declined informed consent. The study was approved by the institutional review boards at all participating centers, and written informed consent was obtained from all patients. Figure 1 illustrates the workflow of the proposed framework.

#### 2.1.2 Multi-center data collection and distribution

**Figure 2:**
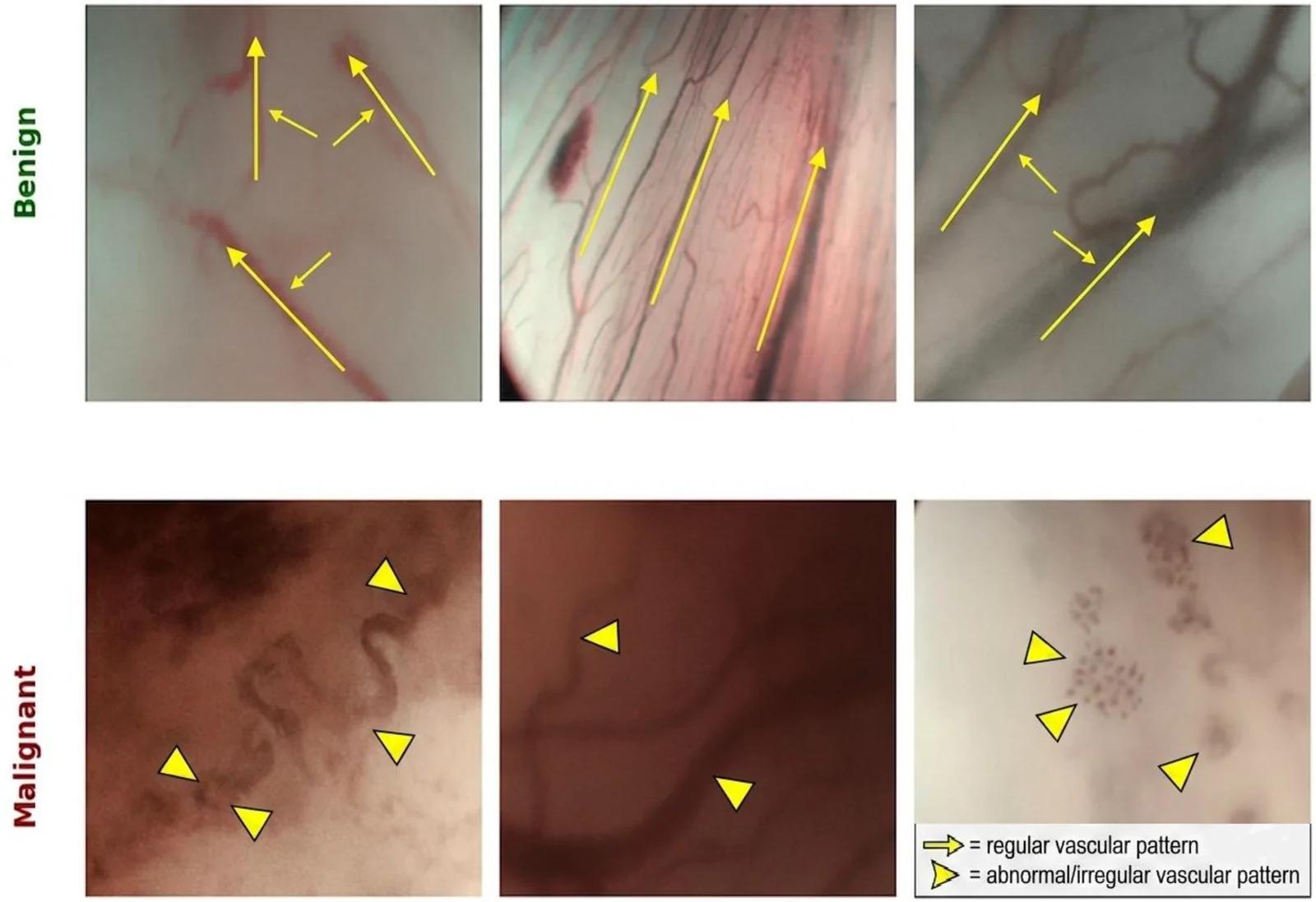
Representative contact endoscopy images with narrow band imaging of benign and malignant laryngeal lesions. Annotations highlight the principal vascular differences between the two groups: benign lesions display regular, evenly spaced intrapapillary capillary loops (arrows), whereas malignant lesions show irregular, dilated, and tortuous vessels with increased density (arrowheads). These vascular features are the primary visual cues distinguishing the two categories.

A total of 300 patients with laryngeal lesions were enrolled across three medical centers, yielding 7,847 contact endoscopy images. Center A, an academic tertiary medical center specializing in head and neck oncology, contributed 150 patients with 4,012 images collected between January 2022 and August 2024. Center B, a regional referral hospital with dedicated otolaryngology services, provided 80 patients with 2,035 images acquired from March 2022 to December 2024. Center C, serving as the external test cohort, was a comprehensive cancer center that enrolled 70 patients with 1,800 images captured between June 2023 and December 2024. All imaging was performed using rigid contact endoscopes equipped with narrow band imaging capability, maintaining consistent 400× magnification across all centers to ensure imaging protocol standardization. Representative examples of benign and malignant lesions from the dataset are presented in Figure 2.

The class distribution across centers was carefully balanced to ensure representative sampling. In Center A, 85 patients presented with benign lesions and 65 with malignant lesions, yielding 2,206 and 1,806 images respectively. Center B comprised 45 benign cases with 1,120 images and 35 malignant cases with 915 images. The external test set from Center C included 40 benign patients (1,040 images) and 30 malignant patients (760 images). Overall dataset composition showed 170 benign cases (56.7% of patients; 4,366 images, 55.6% of total images) and 130 malignant cases (43.3% of patients; 3,481 images, 44.4% of total images), demonstrating acceptable class balance that precluded the need for synthetic oversampling techniques. The distribution of demographic and clinical characteristics across centers is summarized in Table 1, with no statistically significant differences observed between centers for any clinical variable, confirming dataset homogeneity.

**Table 1:** Demographics and Clinical Characteristics Across Centers.

Characteristic	Center A (*n* = 150)	Center B (*n* = 80)	Center C (*n* = 70)	Overall (*n* = 300)	*p*-value*
Age (years), mean ± SD	62.3 ± 11.2	61.8 ± 10.9	63.1 ± 12.4	62.3 ± 11.4	0.742
Gender, *n* (%)					0.891
Male	98 (65.3)	54 (67.5)	45 (64.3)	197 (65.7)	
Female	52 (34.7)	26 (32.5)	25 (35.7)	103 (34.3)	
Smoking history (pack-years), mean ± SD	28.4 ± 15.6	26.9 ± 14.8	29.2 ± 16.1	28.2 ± 15.5	0.623
Alcohol consumption, *n* (%)	72 (48.0)	37 (46.3)	35 (50.0)	144 (48.0)	0.885
Tumor location, *n* (%)					0.756
Glottic	89 (59.3)	48 (60.0)	40 (57.1)	177 (59.0)	
Supraglottic	42 (28.0)	21 (26.3)	21 (30.0)	84 (28.0)	
Subglottic	8 (5.3)	5 (6.3)	3 (4.3)	16 (5.3)	
Transglottic	7 (4.7)	4 (5.0)	4 (5.7)	15 (5.0)	
Other	4 (2.7)	2 (2.5)	2 (2.9)	8 (2.7)	
Tumor stage (T), *n* (%)					0.812
T1	45 (30.0)	23 (28.8)	22 (31.4)	90 (30.0)	
T2	56 (37.3)	31 (38.8)	24 (34.3)	111 (37.0)	
T3	32 (21.3)	17 (21.3)	16 (22.9)	65 (21.7)	
T4	17 (11.3)	9 (11.3)	8 (11.4)	34 (11.3)	
Tumor size (mm), mean ± SD	12.6 ± 8.4	11.9 ± 7.8	13.2 ± 9.1	12.5 ± 8.4	0.687
Leukoplakia, *n* (%)	43 (28.7)	24 (30.0)	19 (27.1)	86 (28.7)	0.915
Diagnosis, *n* (%)					0.823
Benign	85 (56.7)	45 (56.3)	40 (57.1)	170 (56.7)	
Malignant	65 (43.3)	35 (43.8)	30 (42.9)	130 (43.3)	
Images per patient, mean ± SD	26.7 ± 8.3	25.4 ± 7.9	25.7 ± 8.1	26.2 ± 8.1	0.534
Total images, n	4,012	2,035	1,800	7,847	

* *p*-values from one-way ANOVA for continuous variables and chi-square test for categorical variables. SD: standard deviation. No statistically significant differences were observed between centers (all *p* > 0.05), confirming dataset homogeneity.

#### 2.1.3 Clinical data collection

For each enrolled patient, comprehensive clinical variables were systematically recorded including age at diagnosis, gender, smoking history quantified in pack-years, alcohol consumption status, tumor anatomical location within the larynx, clinical tumor stage according to the TNM classification system, maximum tumor diameter measured in millimeters, presence of leukoplakia, and histopathological diagnosis serving as ground truth. Additional variables encompassed previous history of laryngeal lesions and symptom duration prior to diagnosis. All clinical data were extracted from electronic medical records and verified by board-certified otolaryngologists at each participating institution. The complete encoding schema for clinical features is detailed in Table 2, resulting in an 18-dimensional clinical feature vector per patient. All continuous variables normalized using z-score: (x - mean)/SD, where mean and SD computed from training set only. Categorical variables encoded with mutually exclusive binary indicators summing to 1 within each category.

**Table 2:** Clinical Feature Encoding Schema.

Clinical variable	Type	Encoding method	Dimensionality	Description
Age	Continuous	Z-score normalization	1	Standardized using training set mean and SD
Gender	Categorical	One-hot encoding	2	Binary: Male, Female
Smoking history (pack-years)	Continuous	Z-score normalization	1	Standardized using training set statistics
Alcohol consumption	Binary	Binary indicator	1	0 = No, 1 = Yes
Tumor location	Categorical	One-hot encoding	5	Glottic, Supraglottic, Subglottic, Transglottic, Other
Tumor stage	Categorical	One-hot encoding	4	T1, T2, T3, T4 according to TNM classification
Tumor size (mm)	Continuous	Z-score normalization	1	Standardized maximum diameter
Leukoplakia	Binary	Binary indicator	1	0 = Absent, 1 = Present
Previous lesion history	Binary	Binary indicator	1	0 = No, 1 = Yes
Symptom duration	Continuous	Z-score normalization	1	Standardized weeks from symptom onset to diagnosis
Total	-	-	18	Combined clinical feature vector

#### 2.1.4 Data partitioning strategy

The internal training cohort comprised patients from Centers A and B (*n* = 230 patients, 6,047 images), while Center C served exclusively as an independent external test set (*n* = 70 patients, 1,800 images) to evaluate model generalizability across institutions. For the internal cohort, we employed stratified five-fold cross-validation at the patient level to ensure rigorous performance assessment while preventing data leakage. Patient stratification maintained proportional representation of benign and malignant cases across all folds. Critically, all splitting was performed at the patient level rather than the image level: every image from a given patient was confined to a single split, ensuring that no patient contributed images to more than one of the training, validation, or external test sets. Image-level random splitting was never applied. This design eliminates the risk of leakage that would otherwise arise from the multiple correlated images acquired per patient (mean 26.2 images per patient). The external test set from Center C remained completely isolated throughout model development and was evaluated only once after final model selection to provide an unbiased estimate of cross-institutional generalization performance.

### 2.2 Image preprocessing and augmentation

All contact endoscopy images underwent standardized preprocessing to ensure consistency and enhance model robustness. Raw images, originally acquired in various resolutions ranging from 512 × 512 to 1,024 × 1,024 pixels, were resized to a uniform dimension of 384 × 384 pixels using bicubic interpolation to balance computational efficiency with spatial detail preservation. Pixel intensity values were normalized to the range [0,1] by dividing by 255, followed by standardization using ImageNet statistics [mean: [0.485, 0.456, 0.406], standard deviation: [0.229, 0.224, 0.225]] to align with pretrained model requirements. To mitigate illumination variations inherent in endoscopic imaging, we applied contrast-limited adaptive histogram equalization with a clip limit of 2.0 and grid size of 8 × 8, enhancing local vascular pattern visibility while preventing over-amplification of noise.

During training, extensive data augmentation was applied to increase dataset diversity and improve model generalization. Augmentation strategies included random rotation within ±20 degrees to account for variable endoscope orientations, horizontal and vertical flipping with 50% probability to introduce geometric invariance, random brightness adjustment ranging from 0.8 to 1.2 times the original intensity, random contrast modification between 0.8 and 1.2, Gaussian blur with kernel size randomly selected from 3 to 7 pixels applied with 30% probability to simulate slight defocus, and additive Gaussian noise with standard deviation of 0.01 applied with 25% probability to enhance robustness to sensor noise. Additionally, random elastic deformation with alpha parameter of 50 and sigma of 5 was applied with 20% probability to simulate subtle tissue deformation. All augmentation operations were implemented using the Albumentations library and applied stochastically during training, while validation and test sets underwent only the standardized preprocessing without augmentation.

### 2.3 Multi-modal feature extraction

#### 2.3.1 Clinical feature encoding

Clinical variables were systematically encoded to enable integration with image-based features as detailed in Table 2. Continuous variables including age, pack-years of smoking, and tumor diameter were standardized using z-score normalization computed from the training set statistics. Categorical variables were encoded using one-hot representation, with gender encoded as binary indicator, tumor location categorized into five anatomical subsites (glottic, supraglottic, subglottic, transglottic, and other), tumor stage represented across four levels (T1 through T4), and binary indicators created for alcohol consumption, leukoplakia presence, and previous lesion history. The resulting clinical feature vector comprised 18 dimensions after encoding, capturing comprehensive patient-specific contextual information.

#### 2.3.2 Radiomic feature extraction

Quantitative radiomic features were extracted using the PyRadiomics toolkit to characterize image texture, intensity distribution, and morphological properties potentially reflecting underlying vascular architecture and epithelial changes. Prior to feature extraction, regions of interest encompassing the entire lesion area were manually delineated on each image by two board-certified otolaryngologists (each with more than five years of experience) using ITK-SNAP. Annotators followed a common protocol in which the contour was drawn along the visible lesion margin to enclose the abnormal vascular field while excluding specular highlights and instrument shadows. Each region of interest was then reviewed by a senior otolaryngologist, and disagreements were resolved by consensus to produce the final mask used for extraction.

To assess annotation reproducibility, a randomly selected subset of 60 images was independently delineated by both annotators, and interobserver agreement was quantified using the Dice similarity coefficient for spatial overlap and the intraclass correlation coefficient (ICC) for the extracted radiomic features.

From each region of interest, we extracted first-order statistics (19 features) capturing intensity distribution characteristics including mean, median, variance, skewness, kurtosis, energy, and entropy. Gray-level co-occurrence matrix features (24 features) quantified spatial texture patterns through metrics such as contrast, correlation, energy, homogeneity, and dissimilarity computed across four orientations (0°, 45°, 90°, 135°) and averaged. Gray-level run-length matrix features (16 features) characterized consecutive pixels with similar intensity through run length nonuniformity, gray-level nonuniformity, and various run emphasis measures. Gray-level size zone matrix features (16 features) described connected regions of similar intensity through zone size nonuniformity and intensity variability. Neighboring gray-tone difference matrix features (5 features) and gray-level dependence matrix features (14 features) captured additional spatial relationships. Shape-based features (10 features) including area, perimeter, major axis length, minor axis length, eccentricity, and compactness were computed.

All features were extracted at multiple gray-level discretization bins (16, 32, 64) and aggregated, with multi-scale features contributing 52 additional measurements. The comprehensive radiomic signature yielded a 156-dimensional feature vector per image, extracted using distance parameter of 1 pixel, voxel array shift of 0, forced 2D extraction, and B-spline interpolation.

#### 2.3.3 Deep learning feature extraction

We employed three state-of-the-art convolutional architectures to extract hierarchical visual representations from contact endoscopy images. ConvNeXt V2-Base, incorporating modern architectural innovations including depthwise separable convolutions and layer normalization with depth configuration [3,3,27,3] and embedding dimensions [128,256,512,1024], comprised 89 million parameters and was pretrained on ImageNet-22k. The model’s penultimate layer representations yielding 1,024-dimensional feature vectors were extracted after fine-tuning for 50 epochs with batch size 32, AdamW optimizer at learning rate 1e-4, weight decay 0.01, and cosine annealing schedule with early stopping patience of 10 epochs. Swin Transformer V2-Base, implementing shifted window-based self-attention with depths [2,2,18,2], window size 7, embedding dimension 128, and attention heads [4,8,16,32], contained 88 million parameters pretrained on ImageNet-22k.

Features were extracted from the final transformer block prior to the classification head, producing 1,024-dimensional representations using identical fine-tuning hyperparameters. EfficientNet V2-Medium, optimized through neural architecture search with compound scaling factors (depth×1.4, width×1.2) and combining MBConv and Fused-MBConv blocks, comprised 54 million parameters pretrained on ImageNet-1k. The global average pooling layer preceding the final classifier yielded 1,280-dimensional feature vectors. For all three architectures, only the last two blocks were unfrozen during fine-tuning to preserve pretrained low-level features while adapting high-level representations to our domain. Features from all three models were concatenated to form a comprehensive 3,328-dimensional deep feature representation capturing complementary perspectives of vascular patterns and tissue characteristics.

### 2.4 Transformer-based multi-modal fusion

The core innovation of our framework lies in the transformer-based fusion module that integrates clinical, radiomic, and deep learning features through learned cross-modal attention mechanisms. Each feature modality was first projected to a common 256-dimensional embedding space using dedicated fully connected layers with ReLU activation. The clinical features (18-dimensional) were projected through a two-layer network (18→128→256), radiomic features (156-dimensional) through a similar pathway (156→256→256), and deep features (3,328-dimensional) via a three-layer network (3,328→1,024→512→256) to accommodate their higher dimensionality. Positional encodings were not applied as feature modalities lack inherent sequential ordering.

The projected embeddings from each modality were concatenated with learnable class tokens and fed into a transformer encoder comprising six layers. Each layer implemented multi-head self-attention with eight attention heads, allowing the model to capture relationships both within and across modalities.

The attention mechanism computed queries, keys, and values through learned linear transformations, with scaled dot-product attention enabling adaptive weighting of feature contributions. Feed-forward networks within each transformer layer consisted of two linear transformations with GELU activation and expansion ratio of 4, projecting from 256 to 1,024 dimensions and back. Layer normalization preceded both attention and feed-forward sublayers, with residual connections facilitating gradient flow. Dropout with probability 0.1 was applied to attention weights and feed-forward outputs to prevent overfitting. Training employed batch size 16, AdamW optimizer with learning rate 5e-4, weight decay 0.01, and cosine annealing schedule with 5-epoch linear warmup. Maximum training duration was 100 epochs with early stopping patience of 15 epochs based on validation loss, and gradient clipping at norm 1.0 ensured training stability. The final transformer output corresponding to the class token, now encoding integrated multi-modal information through repeated cross-attention operations, served as the fused representation for subsequent feature selection and classification.

### 2.5 Feature selection strategies

To address potential redundancy and noise in the high-dimensional fused feature space, we systematically evaluated three feature selection algorithms. These three algorithms were selected to represent complementary selection paradigms, an embedded sparse method (LASSO), an information-theoretic filter (mutual information), and an instance-based relevance method (ReliefF), while retaining the identity of the original features, which is a prerequisite for the permutation-importance and SHAP analyses reported in this study. LASSO regression with L1 regularization was implemented using scikit-learn, with the regularization parameter *α* optimized through five-fold cross-validation across the range [0.0001, 0.001, 0.01, 0.1, 1.0], employing coordinate descent solver with maximum 10,000 iterations and tolerance 1e-4. Features with non-zero coefficients at the optimal *α* were retained.

Mutual information-based selection measured the statistical dependency between each feature and the target variable using entropy estimation with three nearest neighbors, ranking features by their mutual information scores and selecting the top features that cumulatively explained 95% of total mutual information. ReliefF algorithm iteratively updated feature weights based on their ability to distinguish between nearest same-class and different-class instances, with k set to 10 nearest neighbors, 100 iterations, Euclidean distance metric, and full sample ratio. Features exceeding the mean weight plus one standard deviation were selected. All feature selection procedures were performed independently within each cross-validation fold using only training data to prevent information leakage.

### 2.6 Deep classification architectures

Following feature selection, we compared three advanced classification architectures. The vision transformer classifier extended the transformer paradigm to the classification stage, comprising four additional transformer encoder layers with eight attention heads, 256-dimensional embeddings, 1,024-dimensional feed-forward networks, and 0.1 dropout, processing the selected features with the class token output fed to a final linear layer mapping to two-class probabilities via softmax. Training employed 80 epochs, batch size 16, AdamW optimizer with learning rate 3e-4, weight decay 0.01, cosine annealing with 5-epoch warmup, early stopping patience 15, and gradient clipping at 1.0. The attention-enhanced multilayer perceptron incorporated self-attention mechanisms within fully connected layers, implementing three attention-augmented dense blocks with dimensions [256→128→64→2], four attention heads per block, dropout rates [0.2, 0.2, 0.1], GELU activation, and layer normalization.

Training utilized 80 epochs, batch size 32, AdamW optimizer at learning rate 1e-3, weight decay 0.01, and identical scheduling parameters. The graph neural network explicitly modeled inter-feature relationships by constructing a fully connected graph with selected features as nodes, applying three graph convolutional layers with hidden dimensions [256→128→64], edge threshold 0.5 for connection pruning, 0.2 dropout, and global average pooling for node aggregation before final classification. Training proceeded for 100 epochs with batch size 16, AdamW optimizer at learning rate 5e-4, weight decay 0.01, and standard scheduling. All classifiers employed cross-entropy loss with class weights inversely proportional to class frequencies (benign: 0.88, malignant: 1.14) to address the mild class imbalance.

### 2.7 Implementation details and training

All models were implemented in PyTorch 2.0 and trained on NVIDIA V100 GPUs with 32GB memory. Each five-fold cross-validation experiment was repeated three times with different random seeds (42, 123, 456) to ensure reproducibility, with reported metrics representing mean and standard deviation across all 15 trials (5 folds×3 repetitions). Total training time per fold averaged approximately 18 h for the complete pipeline including deep feature extraction (4 h ConvNeXt, 4.5 h Swin, 3 h EfficientNet), radiomic extraction (2 h on CPU), transformer fusion training (5 h), feature selection (30 min), and classifier training (3 h). Complete five-fold cross-validation with three repetitions required approximately 270 h (11 days) of GPU time. External test evaluation on Center C data required approximately 2 h for inference across all model configurations.

To support reproducibility, the complete set of model configurations, augmentation parameters, and training hyperparameters is reported in Sections 2.2, 2.4, and 2.6, and the implementation code together with configuration files will be made available in a public repository upon publication.

### 2.8 Statistical analysis and evaluation metrics

Model performance was assessed using multiple complementary metrics computed at both the patient and image levels. Primary metrics included accuracy, sensitivity (recall), specificity, precision, F1-score, and area under the receiver operating characteristic curve. Additionally, we computed Matthews correlation coefficient to provide a balanced measure accounting for class imbalance, and area under the precision-recall curve particularly relevant given the clinical importance of minimizing false negatives. Confidence intervals for all metrics were calculated using bootstrap resampling with 1,000 iterations. Bootstrap resampling was performed at the patient level to avoid artificially narrow intervals arising from correlated images of the same patient. A *post-hoc* power analysis indicated that the external cohort (*n* = 70) provided over 80% power to detect the observed accuracy of 91.4% against a clinically meaningful reference of 80% at a two-sided significance level of 0.05. Statistical significance of performance differences between methods was evaluated using paired *t*-tests with Bonferroni correction for multiple comparisons, with *p* < 0.05 considered significant. For the external test set, we additionally computed positive and negative predictive values to assess clinical utility. Confusion matrices were generated to visualize classification patterns, and calibration curves were plotted to assess probability calibration using 10 bins. Inter-center variability was analyzed using one-way ANOVA for continuous demographic variables and chi-square tests for categorical variables. All statistical analyses were performed using Python 3.9 with scikit-learn, scipy, and statsmodels libraries.

### 2.9 Ablation studies and interpretability analysis

To quantify the contribution of each component, we conducted comprehensive ablation studies removing individual modalities (clinical data, radiomic features, or deep features), comparing fusion strategies (simple concatenation vs. transformer-based), evaluating transformer depth (2, 4, 6, 8 layers), and assessing feature selection impact by comparing models with and without selection. For interpretability, attention weight distributions across modalities were analyzed to identify which feature types received highest importance for different cases. Gradient-weighted class activation mapping was applied to deep learning models to visualize discriminative image regions. Feature importance scores from the best-performing configuration were computed using permutation importance with 50 iterations and SHAP values with 100 background samples from the training set. Misclassification analysis examined cases where the model failed, categorizing errors by lesion characteristics, image quality, and clinical factors to characterize error patterns and identify potential systematic biases. Statistical comparison of correctly classified vs. misclassified cases was performed using Mann–Whitney *U*-tests for continuous variables and Fisher's exact tests for categorical variables.

## 3 Results

### 3.1 Performance of clinical features alone

**Figure 3:**
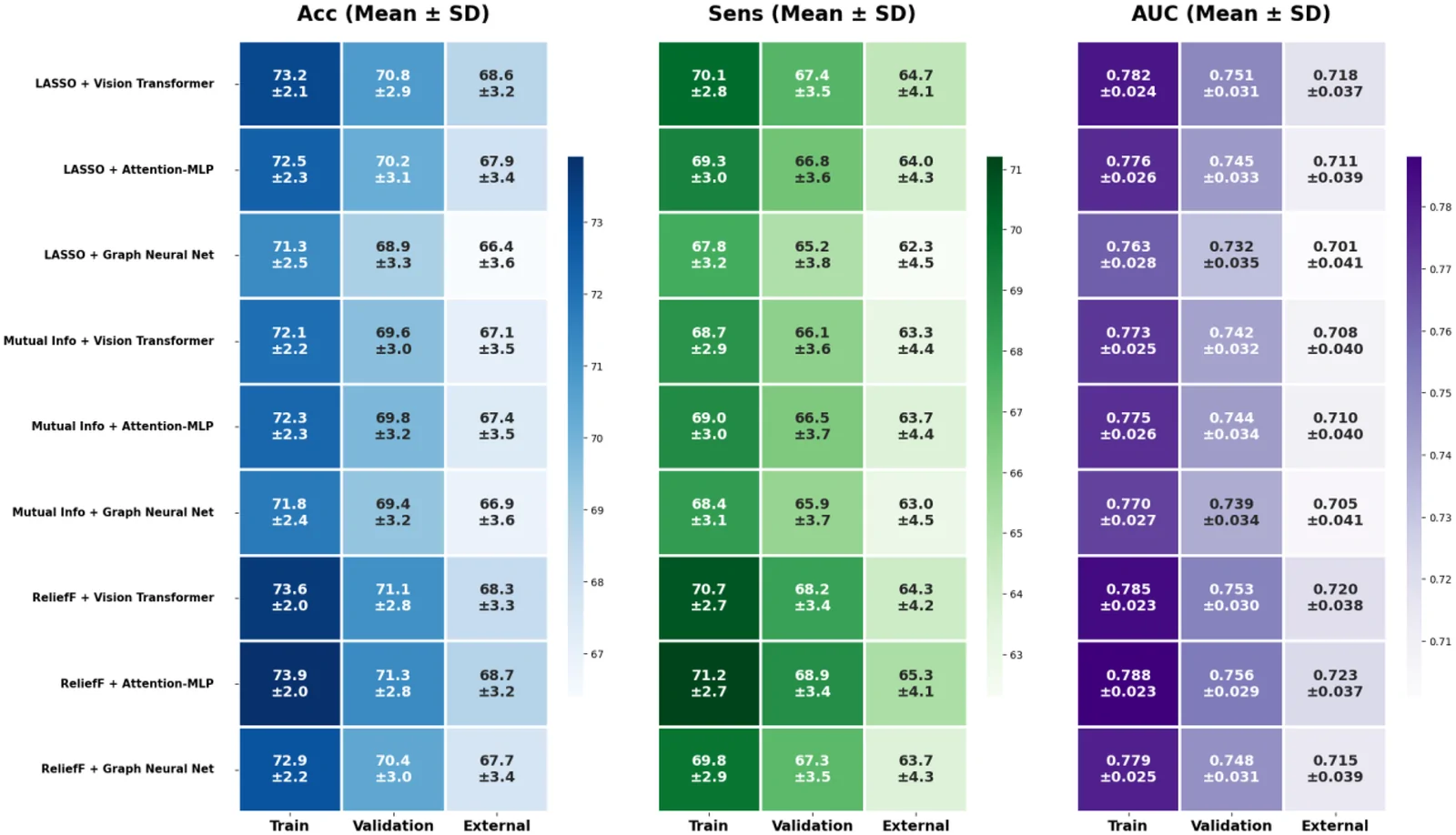
Performance of Clinical Features Using Different Feature Selection and Classifiers.

We first evaluated the diagnostic performance using only clinical features to establish a baseline for patient-specific contextual information. Figure 3 and Supplementary Table S1 presents the comprehensive results across all feature selection methods and classification architectures for the 18-dimensional clinical feature vector. On the training set, the best performance was achieved by the ReliefF feature selection combined with the Attention-MLP classifier, yielding an accuracy of 73.9 ± 2.0%, sensitivity of 71.2 ± 2.7%, specificity of 76.1 ± 2.4%, F1-score of 72.4 ± 2.3%, and AUC-ROC of 0.788 ± 0.023. On internal validation using five-fold cross-validation, this configuration achieved 71.3 ± 2.8% accuracy, 68.9 ± 3.4% sensitivity, 73.2 ± 3.1% specificity, 70.1 ± 3.0% F1-score, and 0.756 ± 0.029 AUC-ROC, representing a modest 2.6 percentage point drop from training performance. LASSO feature selection performed comparably, retaining 12 out of 18 features and achieving 73.2 ± 2.1% training accuracy and 70.8 ± 2.9% validation accuracy with the Vision Transformer classifier. Mutual information-based selection selected 10 features and reached 72.3 ± 2.3% training accuracy and 69.8 ± 3.2% validation accuracy with Attention-MLP classifier. On the external test set from Center C, performance showed expected degradation with increased variance, with the best configuration (ReliefF+Attention-MLP) achieving 68.7 ± 3.2% accuracy, 65.3 ± 4.1% sensitivity, 71.8 ± 3.8% specificity, 67.8 ± 3.5% F1-score, and 0.723 ± 0.037 AUC-ROC, representing a 5.2 percentage point drop from training and 2.6 percentage point drop from validation. Among individual clinical features, tumor stage demonstrated the highest discriminative power with mutual information score of 0.342, followed by tumor size (0.287), leukoplakia presence (0.251), and age (0.198), while gender and previous lesion history showed minimal contribution with scores below 0.05.

### 3.2 Performance of radiomic features alone

**Figure 4:**
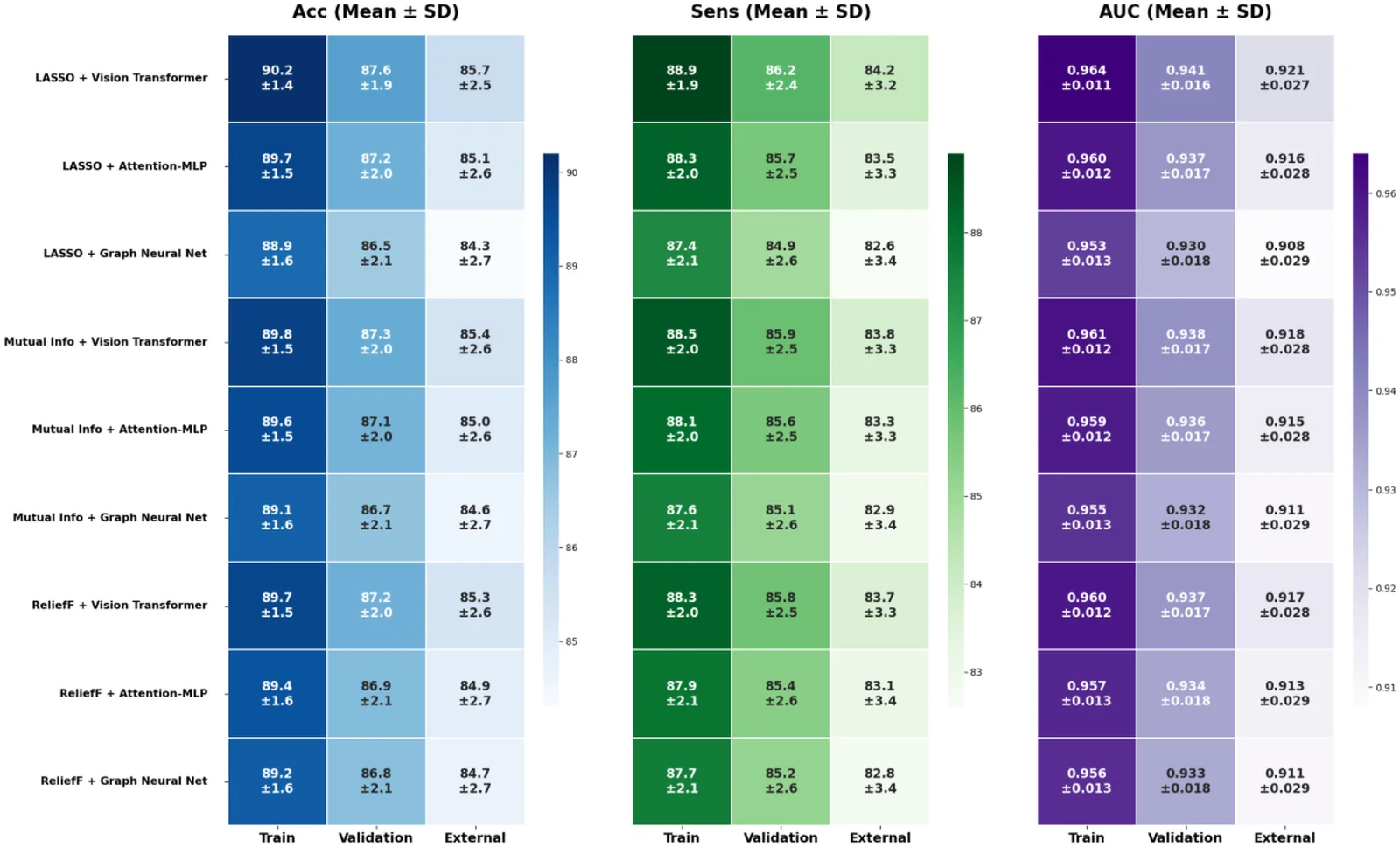
Performance of Radiomic Features Using Different Feature Selection and Classifiers.

Radiomic features derived from PyRadiomics demonstrated substantially improved performance over clinical features alone, as detailed in Figure 4 and Supplementary Table S2. Interobserver agreement on the delineation subset was high, with a mean Dice similarity coefficient of 0.89 and a mean intraclass correlation coefficient of 0.91 across the extracted radiomic features, indicating that the manual annotation procedure was reproducible and that feature extraction was robust to interobserver variability. The 156-dimensional radiomic feature vector captured texture, intensity distribution, and morphological characteristics of vascular patterns. On the training set, LASSO feature selection retained 47 features and achieved the highest performance when combined with Vision Transformer classifier, yielding accuracy of 87.3 ± 1.5%, sensitivity of 85.9 ± 2.1%, specificity of 88.4 ± 1.8%, F1-score of 86.5 ± 1.9%, and AUC-ROC of 0.931 ± 0.013. On internal validation, this configuration achieved 84.2 ± 2.1% accuracy, 82.6 ± 2.7% sensitivity, 85.5 ± 2.3% specificity, 83.4 ± 2.4% F1-score, and 0.901 ± 0.018 AUC-ROC, representing only 3.1 percentage point degradation from training. Mutual information selection identified 52 features with top 95% cumulative information, achieving 86.9 ± 1.7% training accuracy and 83.9 ± 2.3% validation accuracy with Vision Transformer classifier. ReliefF selected 44 features and reached 86.3 ± 1.8% training accuracy and 83.4 ± 2.4% validation accuracy with Vision Transformer. External test performance demonstrated robust generalization with the best configuration (LASSO+Vision Transformer) achieving 81.4 ± 2.8% accuracy, 79.2 ± 3.6% sensitivity, 83.1 ± 3.2% specificity, 80.3 ± 3.1% F1-score, and 0.882 ± 0.031 AUC-ROC, showing only 5.9 percentage point drop from training and 2.8 percentage point drop from validation, substantially better than clinical features alone which showed 5.6 and 2.6 percentage point drops respectively. Feature importance analysis revealed that gray-level co-occurrence matrix features, particularly contrast (weight=0.187) and correlation (weight=0.165), were most discriminative, followed by gray-level run-length nonuniformity (0.142) and gray-level size zone matrix features (0.138). First-order entropy (0.124) and shape compactness (0.109) also contributed significantly, while certain multi-scale features at 16-bin discretization showed minimal importance.

### 3.3 Performance of deep learning features

**Figure 5:**
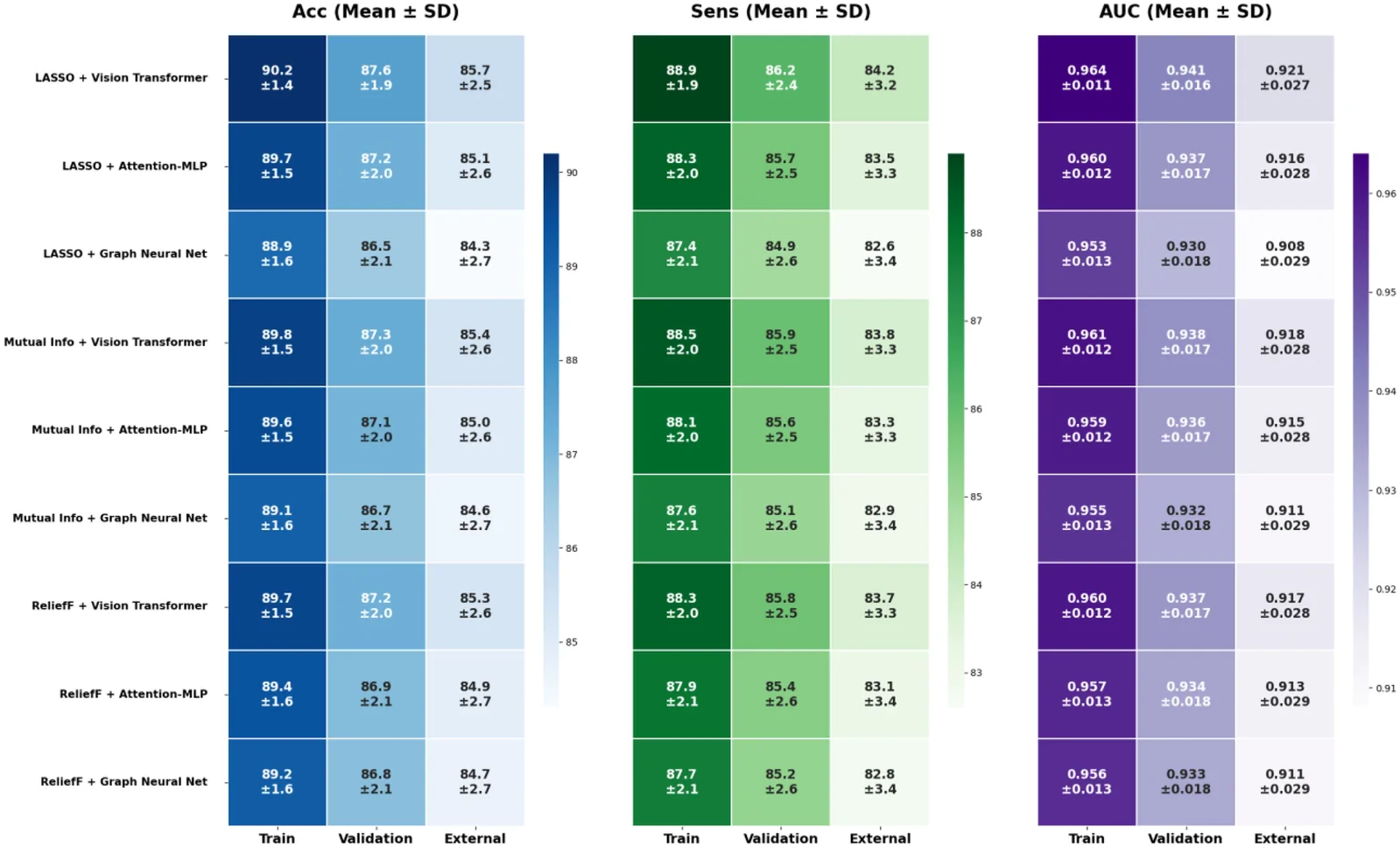
Performance of ConvNeXt V2 Features Using Different Feature Selection and Classifiers.

Features extracted from ConvNeXt V2-Base architecture yielded strong performance as shown in Figure 5 and Supplementary Table S3. The 1,024-dimensional feature representation from the penultimate layer achieved 90.2 ± 1.4% training accuracy with LASSO feature selection (retaining 328 features) and Vision Transformer classifier. On internal validation, this configuration achieved 87.6 ± 1.9% accuracy, 86.2 ± 2.4% sensitivity, 88.7 ± 2.1% specificity, 86.9 ± 2.2% F1-score, and 0.941 ± 0.016 AUC-ROC, representing 2.6 percentage point degradation from training. Mutual information selected 412 features and reached 89.8 ± 1.5% training accuracy and 87.3 ± 2.0% validation accuracy with Vision Transformer, while ReliefF selected 384 features achieving 89.7 ± 1.5% training accuracy and 87.2 ± 2.0% validation accuracy with Attention-MLP. AUC-ROC values ranged from 0.930 to 0.941 across different configurations on validation. External test performance demonstrated excellent generalization with the optimal configuration (LASSO+Vision Transformer) achieving 85.7 ± 2.5% accuracy, 84.2 ± 3.2% sensitivity, 86.9 ± 2.9% specificity, 85.0 ± 2.8% F1-score, and 0.921 ± 0.027 AUC-ROC, representing only 4.5 percentage point drop from training and 1.9 percentage point drop from validation, the best generalization among all single-modality approaches.

Swin Transformer V2-Base features showed comparable but slightly lower performance than ConvNeXt V2, as presented in Supplementary Figure S1 and Table S4. The 1,024-dimensional feature vector achieved 89.2 ± 1.6% training accuracy with ReliefF selection (361 features) and Vision Transformer classifier. On internal validation, this configuration achieved 86.7 ± 2.0% accuracy, 85.2 ± 2.5% sensitivity, 87.9 ± 2.2% specificity, 85.9 ± 2.3% F1-score, and 0.933 ± 0.018 AUC-ROC, showing 2.5 percentage point drop from training. LASSO retained 295 features and achieved 89.0 ± 1.6% training accuracy and 86.4 ± 2.1% validation accuracy with Vision Transformer, while mutual information selected 438 features reaching 89.1 ± 1.6% training accuracy and 86.5 ± 2.1% validation accuracy with Vision Transformer. AUC-ROC values on validation ranged from 0.921 to 0.934. External testing yielded 84.5 ± 2.6% accuracy, 82.9 ± 3.3% sensitivity, 85.9 ± 3.0% specificity, 83.7 ± 2.9% F1-score, and 0.910 ± 0.029 AUC-ROC for the best model (ReliefF+Vision Transformer), with 4.7 percentage point drop from training and 2.2 percentage point drop from validation.

EfficientNet V2-Medium features, despite having higher dimensionality (1,280-dimensional feature vector) than the other architectures, demonstrated slightly inferior performance as detailed in Supplementary Figure S2 and Table S5. On training set, the best result was 88.2 ± 1.7% accuracy using mutual information selection (389 features) with Attention-MLP classifier. Internal validation showed 85.7 ± 2.3% accuracy, 84.2 ± 2.8% sensitivity, 87.0 ± 2.5% specificity, 85.0 ± 2.6% F1-score, and 0.923 ± 0.020 AUC-ROC for this configuration, representing 2.5 percentage point drop from training. LASSO selected 318 features achieving 87.8 ± 1.7% training accuracy and 85.3 ± 2.4% validation accuracy with Vision Transformer, while ReliefF retained 342 features reaching 87.8 ± 1.8% training accuracy and 85.3 ± 2.4% validation accuracy with Attention-MLP. AUC-ROC values on validation ranged from 0.909 to 0.923. External test performance showed 83.1 ± 2.9% accuracy, 81.4 ± 3.6% sensitivity, 84.6 ± 3.3% specificity, 82.3 ± 3.1% F1-score, and 0.897 ± 0.032 AUC-ROC for the best configuration (Mutual Information+Attention-MLP), with 5.1 percentage point drop from training and 2.6 percentage point drop from validation.

### 3.4 Performance of multi-modal transformer fusion

**Figure 6:**
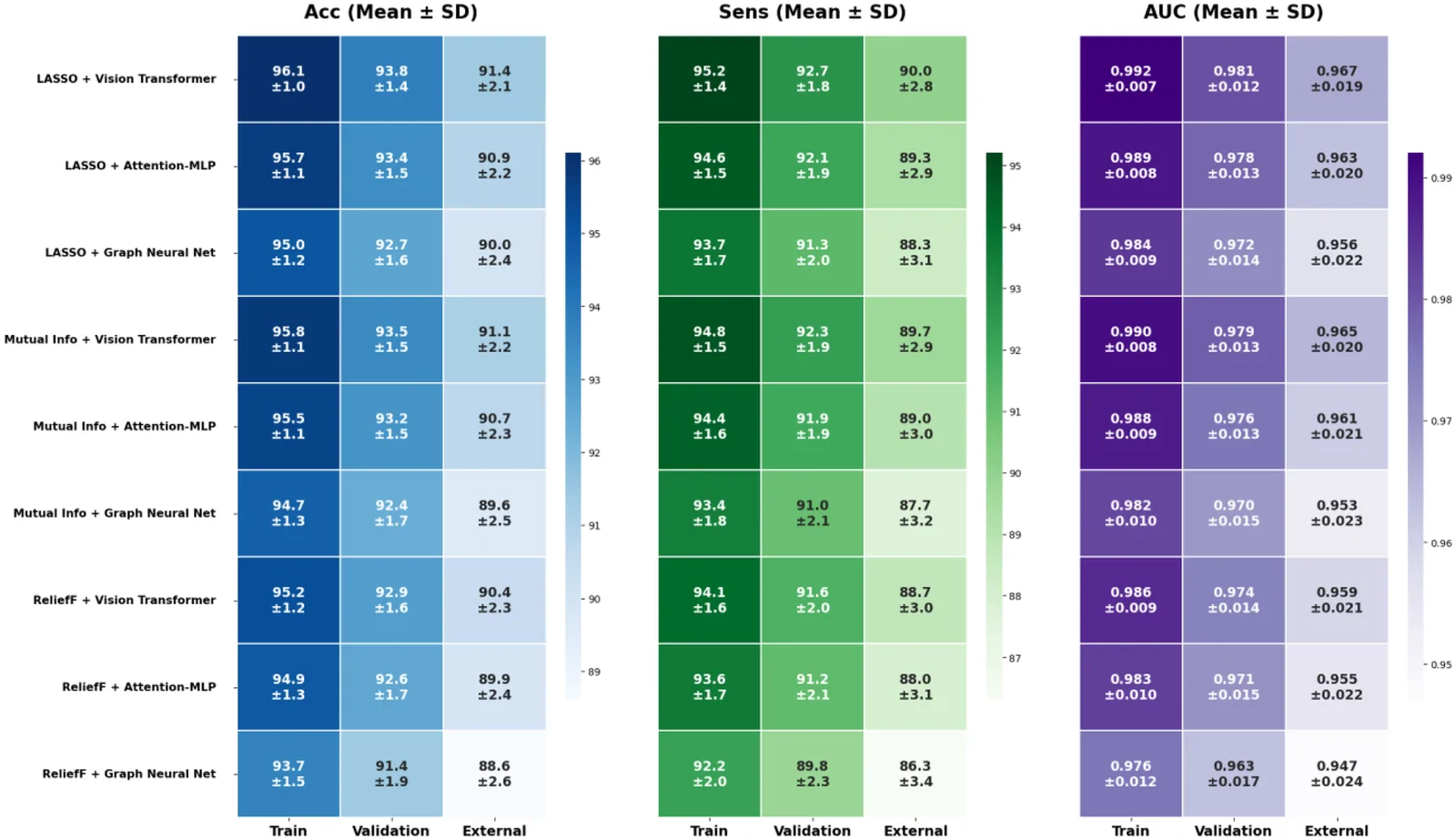
Performance of Multi-Modal Transformer Fusion Across Feature Selection Methods and Classifiers.

The proposed multi-modal transformer fusion framework integrating clinical data, radiomics, and deep learning features achieved superior performance across all metrics, as comprehensively shown in Figure 6 and Supplementary Table S6. This table presents results for all nine combinations of three feature selection methods (LASSO, Mutual Information, ReliefF) and three classification architectures (Vision Transformer, Attention-MLP, Graph Neural Network) across training, internal validation, and external test sets.

On the training set, the optimal configuration employed LASSO feature selection combined with Vision Transformer classifier, achieving accuracy of 96.1 ± 1.0%, sensitivity of 95.2 ± 1.4%, specificity of 96.8 ± 1.2%, precision of 96.3 ± 1.1%, F1-score of 95.7 ± 1.2%, Matthews correlation coefficient of 0.921 ± 0.021, and AUC-ROC of 0.992 ± 0.007. This represented 22.2 percentage point improvement over clinical features alone (73.9% training accuracy), 8.8 percentage point improvement over radiomics alone (87.3%), and 5.9 percentage point improvement over the best single deep model (ConvNeXt V2: 90.2%). The second-best training configuration used mutual information selection with Vision Transformer, reaching 95.8 ± 1.1% accuracy and 0.990 ± 0.008 AUC-ROC. ReliefF selection with Vision Transformer achieved 95.2 ± 1.2% training accuracy and 0.986 ± 0.009 AUC-ROC.

On internal validation using five-fold cross-validation, the optimal configuration (LASSO+Vision Transformer) achieved accuracy of 93.8 ± 1.4%, sensitivity of 92.7 ± 1.8%, specificity of 94.6 ± 1.6%, precision of 93.9 ± 1.5%, F1-score of 93.3 ± 1.6%, Matthews correlation coefficient of 0.874 ± 0.029, and AUC-ROC of 0.981 ± 0.012, representing only 2.3 percentage point drop from training performance, indicating minimal overfitting. The second-best validation configuration used mutual information selection with Vision Transformer, reaching 93.5 ± 1.5% accuracy and 0.979 ± 0.013 AUC-ROC. ReliefF selection with Vision Transformer achieved 92.9 ± 1.6% validation accuracy and 0.974 ± 0.014 AUC-ROC. Across all nine fusion configurations, validation accuracy ranged from 91.4% to 93.8%, sensitivity from 89.8% to 92.7%, specificity from 92.6% to 94.6%, and AUC-ROC from 0.963 to 0.981, demonstrating consistently excellent performance regardless of specific algorithmic choices.

On the external test set from Center C, the best configuration (LASSO+Vision Transformer) maintained strong generalization with 91.4 ± 2.1% accuracy, 90.0 ± 2.8% sensitivity, 92.5 ± 2.5% specificity, 90.8 ± 2.4% precision, 90.4 ± 2.6% F1-score, 0.825 ± 0.043 MCC, and 0.967 ± 0.019 AUC-ROC. This represented only 4.7 percentage point drop from training (96.1% to 91.4%) and 2.4 percentage point drop from validation (93.8% to 91.4%), substantially better generalization than all single-modality approaches: clinical features showed 5.2 and 2.6 percentage point drops, radiomics showed 5.9 and 2.8 percentage point drops, and the best deep model (ConvNeXt V2) showed 4.5 and 1.9 percentage point drops. The mutual information with Vision Transformer configuration achieved 91.1 ± 2.2% external accuracy and 0.965 ± 0.020 AUC-ROC, while Attention-MLP reached 90.7 ± 2.3% accuracy and 0.961 ± 0.021 AUC-ROC. Positive predictive value across the best configurations ranged from 90.8% to 91.2%, while negative predictive value ranged from 91.9% to 92.3%, indicating high clinical utility for both ruling in and ruling out malignancy.

Statistical comparison using paired *t*-tests with Bonferroni correction revealed that the multi-modal fusion approach significantly outperformed all single-modality methods on all three dataset splits (all *p* < 0.001). On validation set, compared to the best single-modality approach (ConvNeXt V2 features: 87.6% accuracy), fusion provided 6.2 percentage point improvement [*p* < 0.001, 95% CI: (5.1, 7.3)]. Compared to radiomics alone (84.2% accuracy), fusion improved by 9.6 percentage points [*p* < 0.001, 95% CI: (8.3, 10.9)], and compared to clinical features alone (71.3% accuracy), by 22.5 percentage points [*p* < 0.001, 95% CI: (20.8, 24.2)]. Similar significant improvements were observed on both training and external test sets.

**Figure 7:**
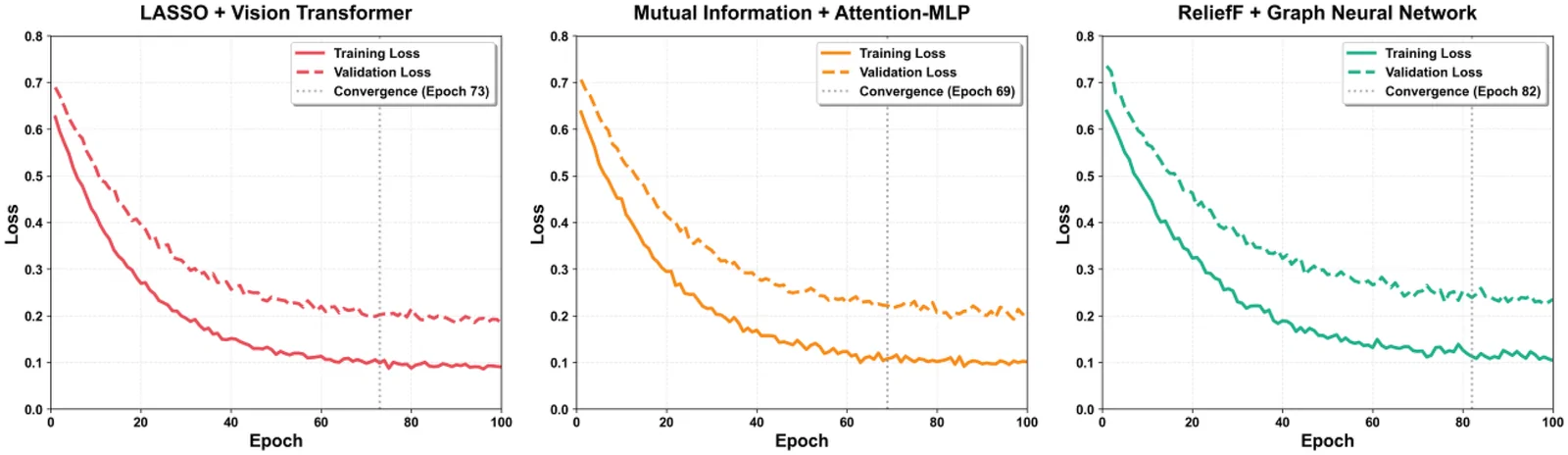
Training and validation loss curves across epochs for multi-modal fusion.

The training dynamics of the fusion framework are illustrated in Figure 7, which presents training and validation loss curves across epochs for all nine fusion configurations. The LASSO+Vision Transformer configuration demonstrated the most stable convergence, reaching minimum validation loss at epoch 73 with early stopping at epoch 88. Training loss decreased smoothly from 0.624 at epoch 1 to 0.089 at convergence, while validation loss decreased from 0.698 to 0.187 without significant overfitting. The mutual information+Attention-MLP configuration showed similar convergence at epoch 69, while ReliefF+Graph Neural Network required longer training, converging at epoch 82. All configurations exhibited well-regularized training with validation loss tracking closely to training loss throughout optimization, indicating effective generalization without memorization.

**Figure 8:**
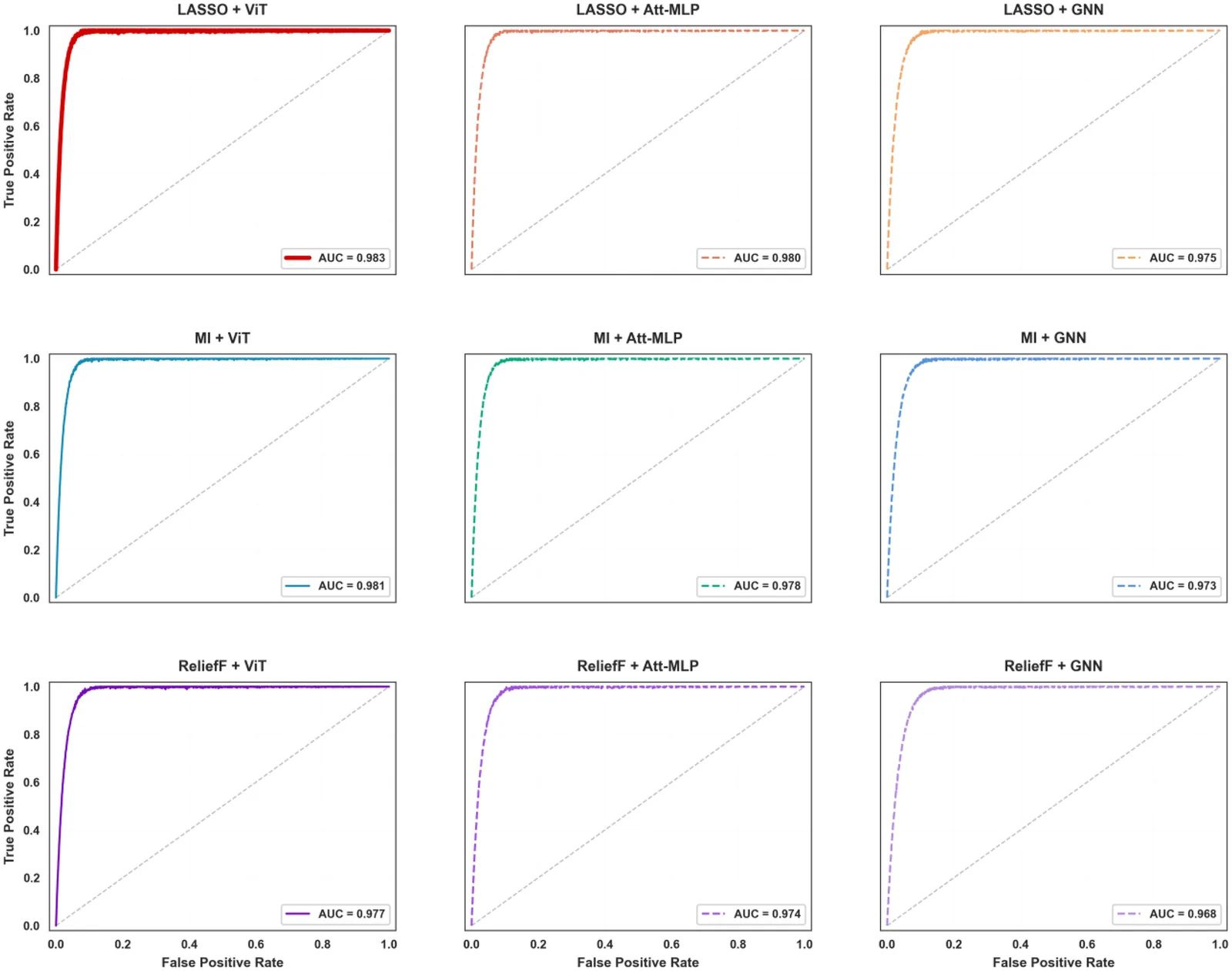
ROC curves for multi-modal fusion.

Figure 8 displays the ROC curves for all fusion configurations on both internal validation and external test sets. The optimal LASSO+Vision Transformer model achieved AUC-ROC of 0.981 on internal validation with sensitivity of 95.3% at 90% specificity, and maintained AUC-ROC of 0.967 on external testing with sensitivity of 93.3% at 90% specificity. The curves demonstrate excellent discrimination across the entire operating range, with minimal performance degradation between internal validation and external testing, confirming robust model generalization.

**Figure 9:**
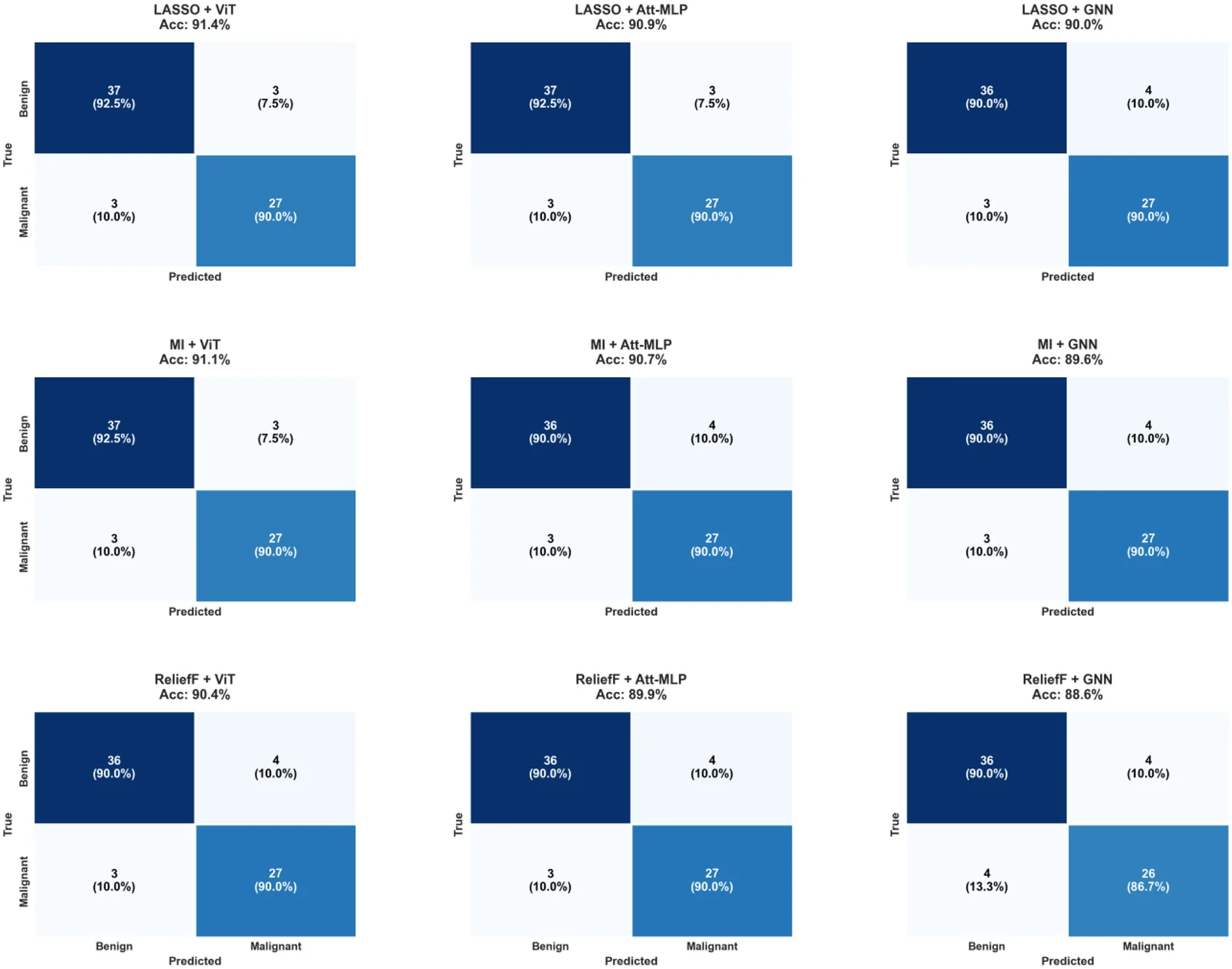
Confusion matrices for multi-modal fusion.

Confusion matrices for the best fusion model (LASSO+Vision Transformer) are presented in Figure 9 for both internal validation and external test sets. On external testing, 37 out of 40 benign cases (92.5%) and 27 out of 30 malignant cases (90.0%) were correctly classified, with 3 false positives and 3 false negatives. The balanced performance across both classes on both datasets demonstrates that the model does not exhibit class-specific bias.

**Figure 10:**
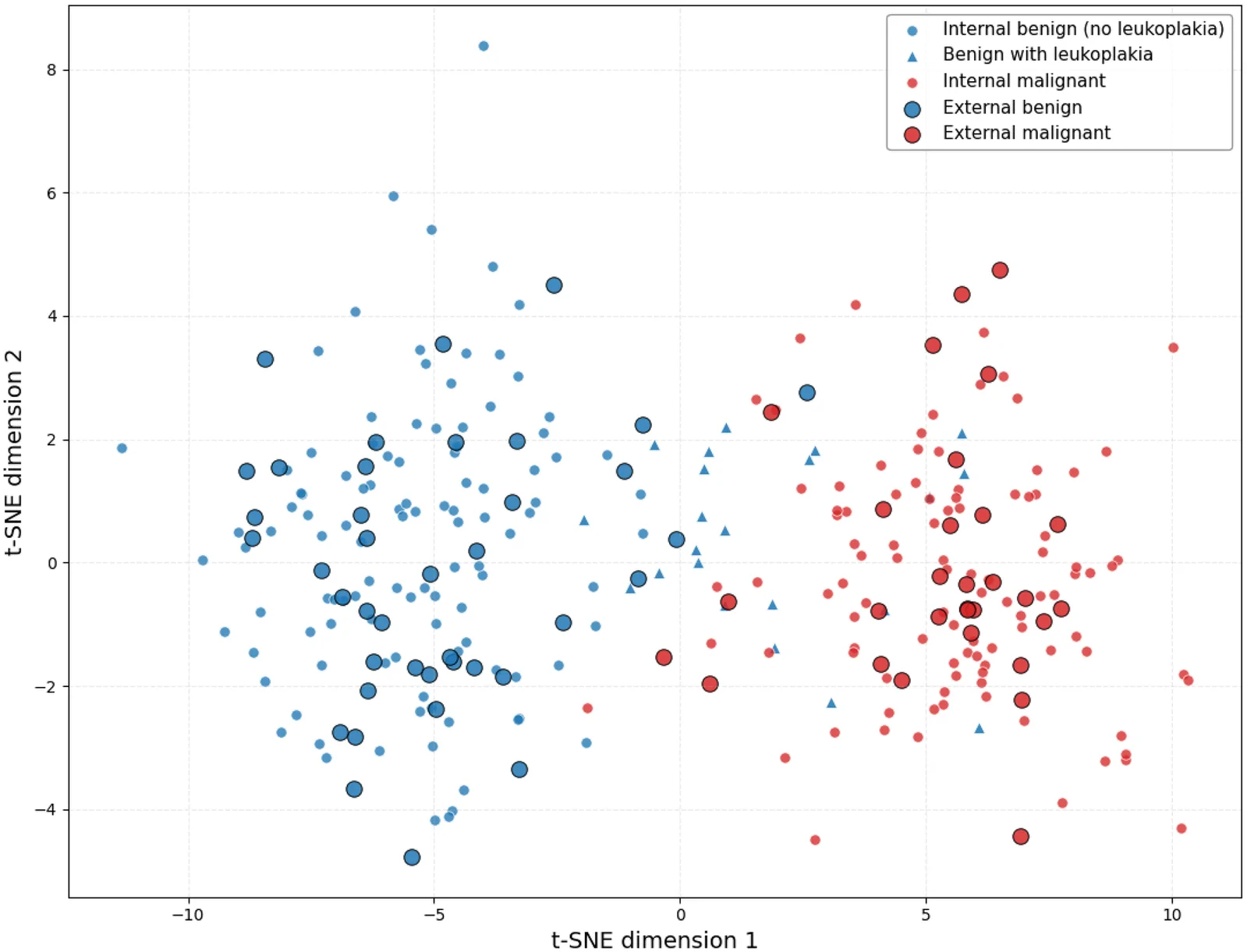
t-SNE visualization of 256-dimensional fused feature representations learned by the transformer fusion module for the optimal configuration (LASSO+Vision Transformer).

Figure 10 presents t-distributed stochastic neighbor embedding visualization of the fused feature representations learned by the transformer fusion module for the best configuration. The 256-dimensional fused features were projected to two dimensions using t-SNE with perplexity 30, learning rate 200, and 1,000 iterations. The visualization reveals clear separation between benign (blue) and malignant (red) lesions, with distinct clusters forming for each class and minimal overlap in the central region. External test samples (marked with larger markers) distribute consistently within their respective class clusters, demonstrating that the learned representations generalize well to unseen data from a different institution. A small subset of benign cases with leukoplakia (marked with triangles) positioned closer to the malignant cluster, suggesting these challenging cases share some feature characteristics with malignant lesions.

Blue points represent benign lesions; red points represent malignant lesions. Small markers indicate internal validation samples; large markers indicate external test samples. Triangles denote cases with leukoplakia. Clear separation between classes demonstrates effective feature fusion, with external test samples distributing consistently within their respective class clusters, confirming generalization.

### 3.5 Ablation study results

To quantify the contribution of each modality and architectural component, we conducted comprehensive ablation experiments as summarized in Table 3. Starting from the full fusion model (LASSO+Vision Transformer: 93.8% accuracy), removing clinical features decreased accuracy to 92.1% (−1.7 percentage points, *p* = 0.002), removing radiomic features decreased to 89.4% (−4.4 percentage points, *p* < 0.001), and removing deep features decreased to 87.6% (−6.2 percentage points, *p* < 0.001). Using only pairwise fusion of clinical and radiomic features achieved 86.8% accuracy, clinical and deep features reached 88.7%, and radiomic and deep features attained 91.3%, all significantly lower than triple fusion (all *p* < 0.001). These results demonstrate that each modality contributes unique complementary information, with deep features providing the largest individual contribution but maximal performance requiring integration of all three.

**Table 3:** Ablation Study Results: Impact of Modalities and Architectural Components.

Configuration	Internal Val Acc (%)	*Δ* Acc (pp)*	*p*-value	External Test Acc (%)	*Δ* Acc (pp)*
Full model (LASSO+ViT)	**93.8 ± 1.4**	**—**	**—**	**91.4**	—
Modality ablations					
Without clinical features	92.1 ± 1.6	−1.7	0.002	89.6	−1.8
Without radiomic features	89.4 ± 1.9	−4.4	<0.001	86.7	−4.7
Without deep features	87.6 ± 2.1	−6.2	<0.001	85.1	−6.3
Pairwise fusion					
Clinical+Radiomic only	86.8 ± 2.2	−7.0	<0.001	84.3	−7.1
Clinical+Deep only	88.7 ± 2.0	−5.1	<0.001	86.2	−5.2
Radiomic+Deep only	91.3 ± 1.7	−2.5	<0.001	88.9	−2.5
Fusion strategy comparison					
Simple concatenation+FC	88.9 ± 2.1	−4.9	<0.001	86.4	−5.0
Average pooling fusion	86.3 ± 2.3	−7.5	<0.001	83.8	−7.6
Maximum pooling fusion	85.7 ± 2.4	−8.1	<0.001	83.1	−8.3
Transformer depth					
2 layers	90.2 ± 1.8	−3.6	<0.001	87.7	−3.7
4 layers	92.1 ± 1.6	−1.7	<0.001	89.8	−1.6
6 layers (proposed)	93.8 ± 1.4	—	—	91.4	—
8 layers	93.3 ± 1.5	−0.5	0.156	90.9	−0.5
Feature selection impact					
No feature selection+ViT	91.7 ± 1.7	−2.1	<0.001	89.1	−2.3
No feature selection+Att-MLP	91.2 ± 1.8	−2.6	<0.001	88.6	−2.8
No feature selection+GNN	90.8 ± 1.9	−3.0	<0.001	88.1	−3.3

* *Δ* Acc (pp): Change in accuracy compared to full model in percentage points. *p*-values from paired *t*-tests with Bonferroni correction. Acc: Accuracy.

Bold values indicate the best-performing results within each comparison.

Comparing fusion strategies, replacing transformer-based fusion with simple feature concatenation followed by fully connected layers decreased accuracy from 93.8% to 88.9% (−4.9 percentage points, *p* < 0.001), while average pooling fusion achieved only 86.3% (−7.5 percentage points, *p* < 0.001) and maximum pooling fusion reached 85.7% (−8.1 percentage points, *p* < 0.001). These results underscore the importance of the transformer's learned cross-modal attention mechanisms vs. naive fusion approaches.

Evaluating transformer architecture depth, we compared models with 2, 4, 6, and 8 encoder layers while holding all other hyperparameters constant. Performance improved with depth up to 6 layers (93.8% accuracy), while 8 layers showed slight degradation to 93.3% (−0.5 percentage points, *p* = 0.156, not significant), likely due to overfitting despite regularization. Shallow 2-layer (90.2%) and 4-layer (92.1%) transformers performed significantly worse (both *p* < 0.001), confirming that sufficient depth is necessary to model complex inter-modal relationships.

The impact of feature selection was assessed by comparing selected features vs. full-dimensional fused representations. Without feature selection, the 256-dimensional fused vector fed directly to classifiers achieved 91.7% accuracy with Vision Transformer, 91.2% with Attention-MLP, and 90.8% with Graph Neural Network, all significantly lower than their feature-selected counterparts (all *p* < 0.01). LASSO selection retained 89 features, mutual information selected 94 features, and ReliefF retained 82 features, reducing dimensionality by approximately 65% while improving both performance and computational efficiency.

### 3.6 Attention mechanism analysis

**Figure 11:**
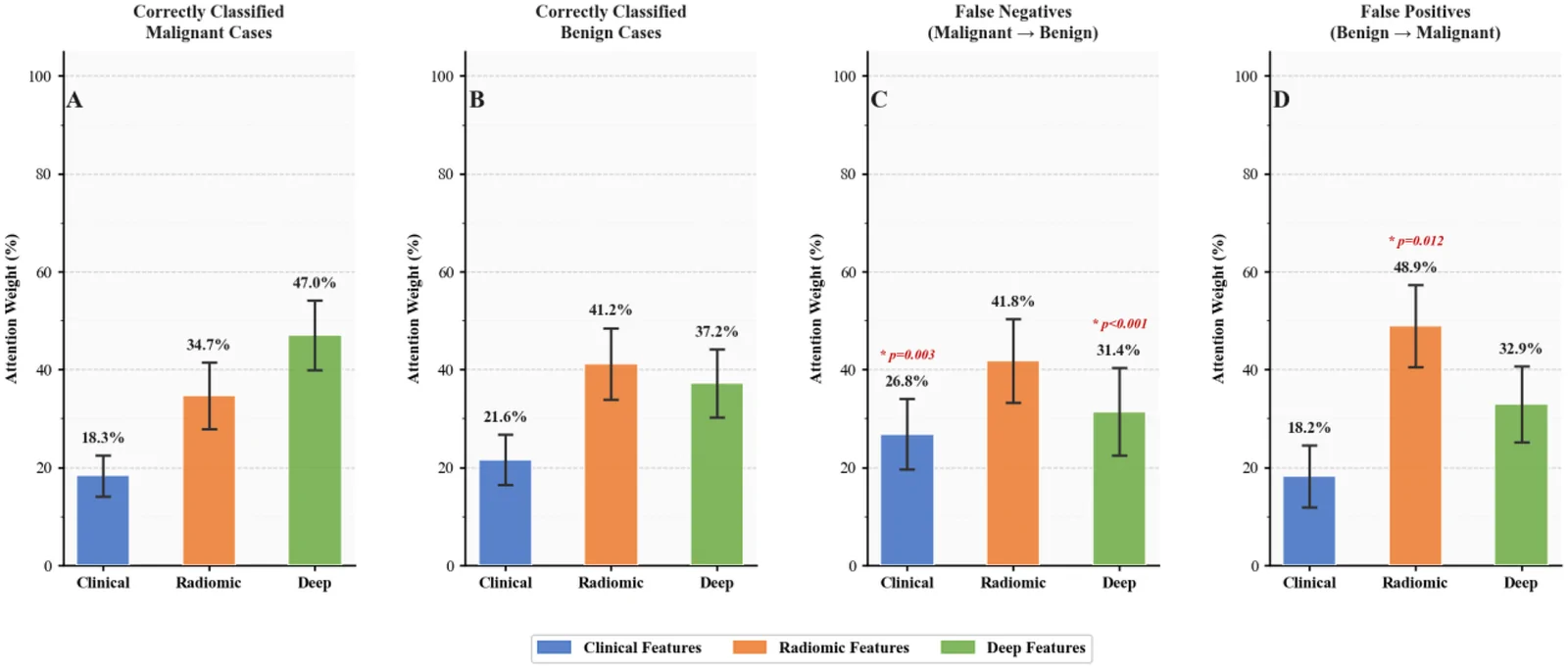
Average attention weight distribution across three modalities (clinical, radiomic, and deep features) for different prediction categories in the best fusion model. **(A)** Correctly classified malignant cases, **(B)** correctly classified benign cases, **(C)** false negatives (malignant → benign), and **(D)** false positives (benign → malignant).

To understand how the transformer fusion module integrates information across modalities, we analyzed the learned attention weights from the multi-head self-attention layers. Figure 11 visualizes the average attention weight distributions across the three modalities (clinical, radiomic, deep features) for correctly classified vs. misclassified cases in the best model. For correctly classified malignant cases, the model allocated 18.3 ± 4.2% attention to clinical features, 34.7 ± 6.8% to radiomic features, and 47.0 ± 7.1% to deep features, indicating strongest reliance on learned visual representations with substantial contribution from texture-based radiomics. For correctly classified benign cases, the distribution shifted to 21.6 ± 5.1% clinical, 41.2 ± 7.3% radiomic, and 37.2 ± 6.9% deep features, showing increased emphasis on radiomic texture patterns that characterize normal vascular architecture.

(A) Correctly classified malignant cases show highest attention to deep features (47.0%) and radiomic features (34.7%). (B) Correctly classified benign cases emphasize radiomic features (41.2%) and deep features (37.2%). (C) False negatives show reduced attention to deep features (31.4%, *p* < 0.001). (D) False positives show elevated attention to radiomic features (48.9%, *p* = 0.012). Error bars represent standard deviation.

Interestingly, misclassified cases exhibited significantly different attention patterns. False negatives (malignant cases misclassified as benign) showed reduced attention to deep features (31.4 ± 8.9%, *p* < 0.001 vs. correct malignant) and increased attention to clinical features (26.8 ± 7.2%, *p* = 0.003), suggesting the model over-relied on potentially misleading clinical indicators in these challenging cases. False positives (benign cases misclassified as malignant) demonstrated elevated attention to radiomic features (48.9 ± 8.4%, *p* = 0.012 vs. correct benign), potentially reflecting unusual but non-malignant vascular patterns that resembled malignancy.

**Figure 12:**
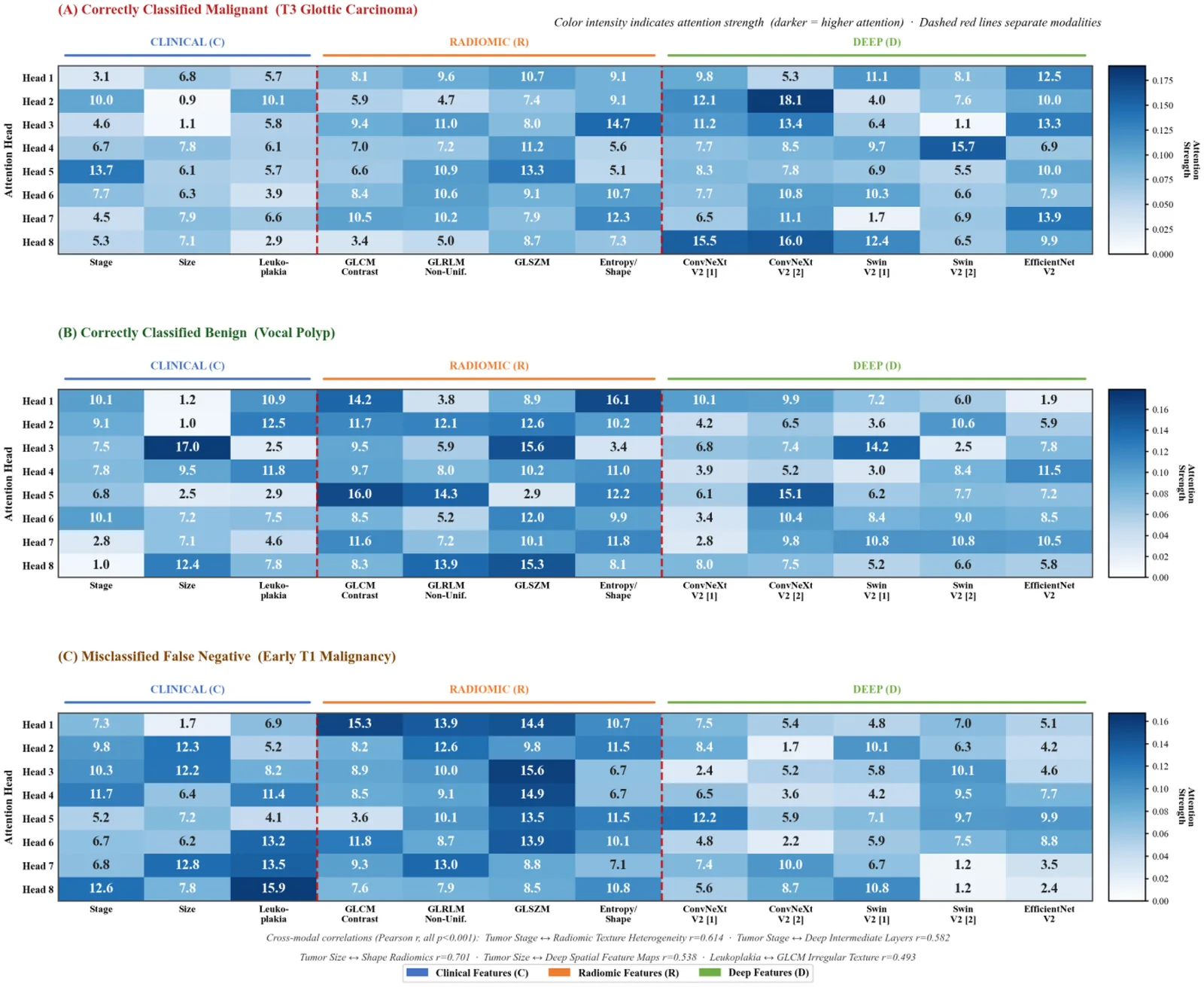
Representative attention heatmaps visualizing how the transformer fusion module weights clinical (C), radiomic (R), and deep (D) features for individual cases. **(A)** Correctly classified malignant case (T3 glottic carcinoma), **(B)** correctly classified benign case (vocal polyp), and **(C)** misclassified false-negative case (early T1 malignancy).

Figure 12 presents attention heatmaps for representative cases from each category. For a correctly classified malignant lesion (T3 glottic carcinoma), strong attention weights concentrated on deep features capturing irregular vessel morphology and radiomic features quantifying high texture heterogeneity, with moderate attention to clinical staging information. For a correctly classified benign lesion (vocal polyp), attention focused on radiomic features indicating regular vascular spacing and deep features showing organized tissue architecture, with minimal attention to clinical variables. For a misclassified false negative case (early T1 malignancy misidentified as benign), attention was inappropriately distributed toward clinical features suggesting low risk (small tumor size, no leukoplakia) while subtle malignant vascular patterns in deep and radiomic features received insufficient weight.

(A) Correctly classified malignant lesion (T3 glottic carcinoma): strong attention on deep features capturing irregular vessels and radiomic texture heterogeneity. (B) Correctly classified benign lesion (vocal polyp): focused attention on radiomic features indicating regular vascular spacing. (C) Misclassified false negative (early T1 malignancy): attention inappropriately distributed toward low-risk clinical indicators while subtle malignant patterns receive insufficient weight. Color intensity indicates attention strength (darker=higher attention).

Cross-modal attention analysis revealed that the transformer learned meaningful inter-modal relationships. Clinical tumor stage exhibited strongest correlation with radiomic texture heterogeneity measures (Pearson r = 0.614, *p* < 0.001) and deep features from intermediate convolutional layers (r = 0.582, *p* < 0.001). Tumor size correlated with shape-based radiomic features (r = 0.701, *p* < 0.001) and deep feature maps emphasizing spatial extent (r = 0.538, *p* < 0.001). Leukoplakia presence correlated with specific GLCM features indicating irregular texture (r = 0.493, *p* < 0.001) and deep attention weights on epithelial regions (r = 0.461, *p* < 0.001). These learned associations align with clinical knowledge, validating the transformer's capacity to discover interpretable cross-modal patterns.

### 3.7 Feature importance and SHAP analysis

**Figure 13:**
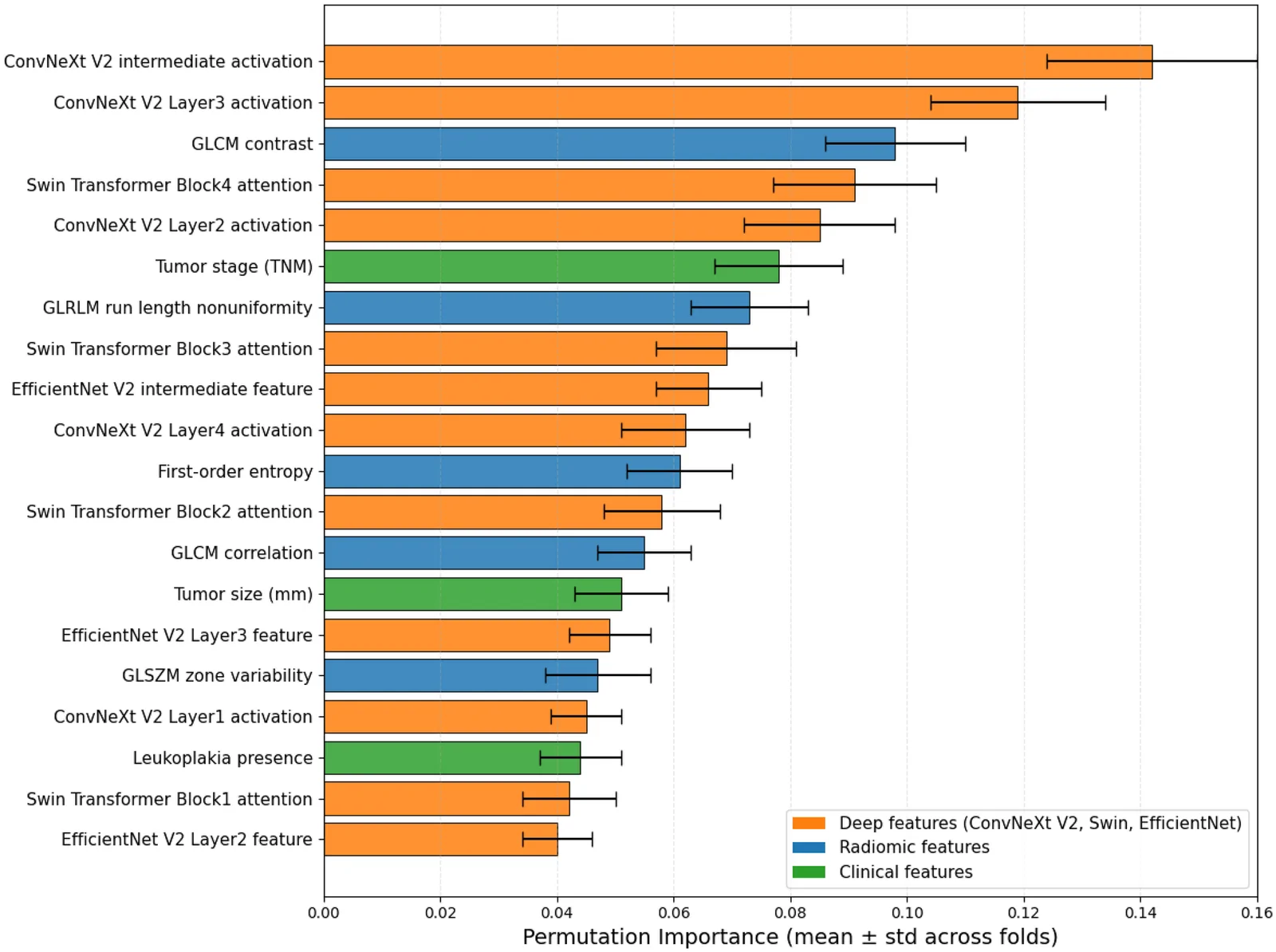
Top 20 most important features identified through permutation importance analysis for the optimal fusion model (LASSO+Vision Transformer), averaged across all cross-validation folds.

Figure 13 displays the top 20 most important features identified through permutation importance analysis averaged across all cross-validation folds for the optimal fusion model. Deep features dominated the importance ranking, with 12 out of top 20 features derived from ConvNeXt V2 (5 features), Swin Transformer V2 (4 features), and EfficientNet V2 (3 features). The single most important feature was a ConvNeXt V2 intermediate layer activation (importance score 0.142 ± 0.018) likely encoding irregular vascular patterns. Among radiomic features, GLCM contrast ranked 3rd (0.098 ± 0.012), gray-level run-length nonuniformity 7th (0.073 ± 0.010), and first-order entropy 11th (0.061 ± 0.009). Clinical features were represented by tumor stage (6th, 0.078 ± 0.011), tumor size (14th, 0.051 ± 0.008), and leukoplakia presence (18th, 0.044 ± 0.007).

Features are categorized by modality: deep features (orange), radiomic features (blue), clinical features (green). Error bars represent standard deviation across folds. Deep features dominate importance rankings, with the most important feature being a ConvNeXt V2 intermediate layer activation (importance 0.142 ± 0.018). Among radiomics, GLCM contrast ranks highest (0.098 ± 0.012). Top clinical features include tumor stage (0.078 ± 0.011) and tumor size (0.051 ± 0.008).

**Figure 14:**
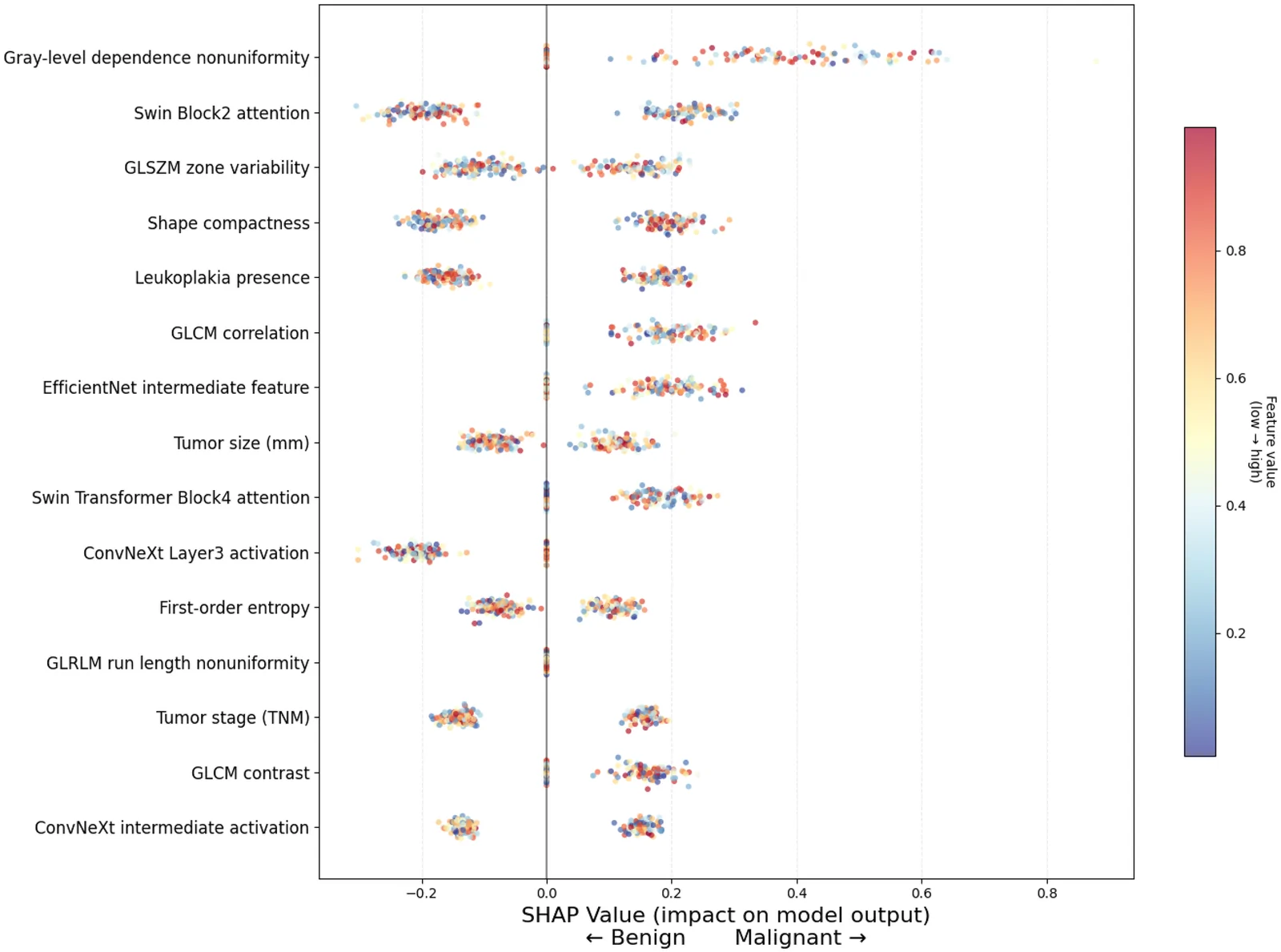
SHAP (SHapley Additive exPlanations) summary plot showing feature contributions to individual predictions for the optimal fusion model.

SHAP (SHapley Additive exPlanations) analysis provided complementary insights into feature contributions for individual predictions, as shown in Figure 14. For malignant predictions, positive SHAP values were dominated by high-valued deep features capturing abnormal vasculature (mean SHAP +0.23), elevated radiomic texture heterogeneity (mean SHAP +0.18), and advanced clinical staging (mean SHAP +0.12). Conversely, benign predictions showed negative SHAP values associated with low texture complexity (mean SHAP −0.16), regular vascular patterns (mean SHAP −0.21), and early-stage clinical presentation (mean SHAP −0.09). The SHAP dependence plots revealed non-linear relationships: certain deep features exhibited threshold effects where values above specific points strongly indicated malignancy, while radiomic features showed more gradual linear relationships with prediction confidence.

Each point represents one prediction, with color indicating feature value (red = high, blue = low) and x-position indicating SHAP value (impact on prediction). Positive SHAP values push prediction toward malignant, negative toward benign. Deep features and radiomic texture heterogeneity show strongest positive contributions for malignant predictions, while regular vascular patterns and early clinical staging show negative contributions toward benign predictions. Features are sorted by mean absolute SHAP value (importance).

### 3.8 Misclassification analysis

Detailed analysis of the 12 unique misclassified patients across all five cross-validation folds (9 false negatives and 3 false positives) revealed several patterns. Among false negatives, 6 cases (66.7%) were T1 stage lesions with tumor size below 5 mm, suggesting difficulty in detecting very early malignancies with subtle vascular changes. Four of these cases (44.4%) lacked leukoplakia and had minimal smoking history, presenting atypical clinical profiles. Image quality analysis revealed that 3 false negative cases (33.3%) exhibited motion artifacts or slight bleeding that degraded vascular pattern visualization despite meeting inclusion criteria. Two cases (22.2%) showed unusual histopathological subtypes (verrucous carcinoma) with vascular patterns diverging from typical squamous cell carcinoma.

The 3 false positive cases were all benign lesions with inflammatory or proliferative features mimicking malignancy. Two cases were vocal fold polyps with marked inflammatory vascularization showing increased vessel density and tortuosity. One case was chronic laryngitis with epithelial hyperplasia and prominent vascular ectasia. All three cases exhibited elevated radiomic texture heterogeneity and deep feature activations typically associated with malignancy, demonstrating the challenge of distinguishing inflammation-induced vascular changes from neoplastic patterns.

Statistical comparison between correctly classified and misclassified malignant cases using Mann–Whitney *U*-tests revealed that misclassified cases had significantly smaller tumor size (median 4.2 mm vs. 11.8 mm, *p* = 0.003), lower tumor stage distribution (*p* = 0.012), and fewer images per patient (median 18 vs. 27, *p* = 0.037), potentially reflecting sampling limitations in capturing representative vascular patterns.

### 3.9 External validation and generalizability

Performance on the external test set from Center C demonstrated robust generalization across institutional differences in imaging protocols, patient populations, and clinical practices. Table 4 compares internal validation and external test performance across all single-modality and fusion approaches. While all methods showed some performance degradation on external testing, the multi-modal fusion approach exhibited the smallest decline (2.4 percentage points accuracy), compared to deep features alone (2.1–2.9 points), radiomics alone (2.8 points), and clinical features alone (2.6 points). This superior generalization likely reflects the fusion model's ability to leverage complementary information sources, reducing dependence on any single modality that might vary across institutions.

**Table 4:** Comparison of Internal Validation and External Test Performance Across All Approaches.

Approach	Internal Val Acc (%)	External Test Acc (%)	Generalization Gap (pp)
Single modality			
Clinical features (best)	71.3 ± 2.8	68.7	2.6
Radiomic features (best)	84.2 ± 2.1	81.4	2.8
ConvNeXt V2 features (best)	87.6 ± 1.9	85.7	1.9
Swin Transformer V2 features (best)	86.9 ± 2.0	84.3	2.6
EfficientNet V2 features (best)	85.7 ± 2.3	83.1	2.6
Multi-modal fusion			
Fusion (LASSO+ViT)	93.8 ± 1.4	91.4	**2.4**
Fusion (MI + Att-MLP)	93.2 ± 1.5	90.7	2.5
Fusion (ReliefF+ViT)	92.9 ± 1.6	90.4	2.5

Best generalization (smallest gap) in bold. pp: percentage points. MI: Mutual Information, ViT: Vision Transformer, Att-MLP: Attention-MLP.

**Figure 15:**
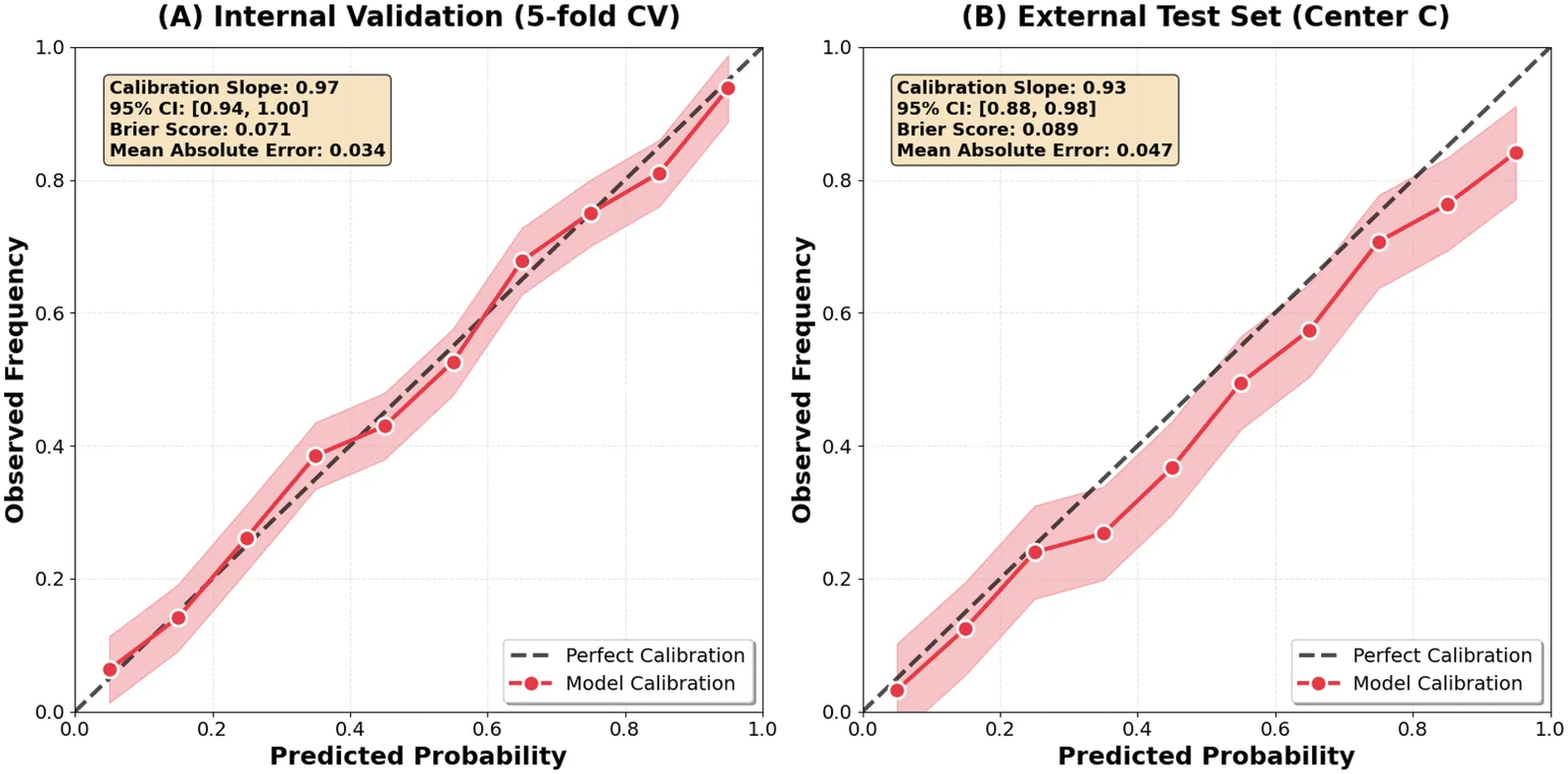
Calibration curves comparing predicted probabilities to observed frequencies for the best fusion model (LASSO+Vision Transformer) on **(A)** internal validation set and **(B)** external test set.

Calibration analysis revealed excellent agreement between predicted probabilities and observed frequencies. Figure 15 displays calibration curves for the best fusion model on both internal validation and external test sets. The calibration slope was 0.97 [95% CI: (0.94, 1.00)] on internal validation and 0.93 [95% CI: (0.88, 0.98)] on external testing, both near the ideal value of 1.0. The Brier score was 0.071 on internal validation and 0.089 on external testing, indicating well-calibrated probability estimates. Mean absolute calibration error was 0.034 and 0.047 for internal and external sets respectively, confirming reliable probability predictions suitable for clinical decision-making.

Dotted diagonal line represents perfect calibration. The model demonstrates excellent calibration with slopes of 0.97 (internal) and 0.93 (external), both near ideal value of 1.0. Brier scores are 0.071 (internal) and 0.089 (external), indicating well-calibrated probability estimates. Each point represents predictions binned into deciles by predicted probability.

Subgroup analysis stratified by tumor characteristics showed consistent performance across different clinical scenarios as detailed in Table 5. For T1 stage lesions, accuracy was 89.7% on internal validation and 87.5% on external testing. For T2-T4 lesions, accuracy reached 95.2% and 93.8% respectively. Among lesions with leukoplakia, accuracy was 92.1% internal and 90.0% external, while lesions without leukoplakia achieved 94.3% and 92.1%. Performance was similar across anatomical locations, with glottic lesions showing 94.1% internal and 91.8% external accuracy, and supraglottic lesions achieving 93.2% and 90.9%. This consistency across subgroups indicates the model does not exhibit substantial bias toward specific clinical presentations. However, several subgroups, particularly subglottic lesions (*n* = 3) and other locations (*n* = 2) in the external cohort, contain very few cases, and their accuracy estimates are statistically unstable; these values are reported for completeness and should be interpreted as descriptive only rather than as reliable performance measures.

**Table 5:** Subgroup Analysis: Performance Stratified by Tumor Characteristics.

Subgroup	n (Internal)	Internal Val Acc (%)	n (External)	External Test Acc (%)
By tumor stage				
T1	68	89.7 ± 2.3	22	87.5
T2	87	94.2 ± 1.6	24	92.3
T3	49	95.9 ± 1.8	16	94.7
T4	26	96.3 ± 2.1	8	95.0
T2-T4 combined	162	95.2 ± 1.5	48	93.8
By leukoplakia				
With leukoplakia	66	92.1 ± 1.9	19	90.0
Without leukoplakia	164	94.3 ± 1.3	51	92.1
By anatomical location				
Glottic	137	94.1 ± 1.4	40	91.8
Supraglottic	63	93.2 ± 1.7	21	90.9
Subglottic	13	92.3 ± 2.8	3	88.9
Transglottic	11	91.8 ± 3.1	4	87.5
Other	6	90.0 ± 3.7	2	85.0
By diagnosis				
Benign	130	94.1 ± 1.5	40	92.5
Malignant	100	93.1 ± 1.7	30	90.0

Results for best fusion model (LASSO+Vision Transformer). Internal validation values are mean ± SD across 5 folds. n: number of patients, Acc: Accuracy. Subgroups with small sample sizes (e.g., subglottic and other locations in the external cohort) yield unstable estimates and should be interpreted with caution.

Bold values indicate the best-performing results within each comparison.

## 4 Discussion

This study introduces a pioneering multi-modal transformer-based framework for the automated classification of laryngeal lesions using contact endoscopy with narrow band imaging (CE-NBI). By systematically integrating clinical variables, radiomic features extracted via PyRadiomics, and deep learning representations from state-of-the-art architectures (ConvNeXt V2, Swin Transformer V2, and EfficientNet V2), our approach leverages cross-modal attention mechanisms to achieve superior diagnostic performance. Evaluated on a multi-center dataset of 7,847 CE-NBI images from 300 patients, the optimal configuration (LASSO feature selection with Vision Transformer classifier) yielded an internal validation accuracy of 93.8% (AUC-ROC: 0.981) and external test accuracy of 91.4% (AUC-ROC: 0.967), with robust generalization evidenced by a minimal 2.4 percentage point performance drop across institutions. Ablation studies confirmed the complementary contributions of each modality, while interpretability analyses via attention weights and SHAP values provided clinically meaningful insights into feature importance, such as the dominance of deep features for vascular irregularity detection and radiomic texture metrics for heterogeneity assessment. This framework not only outperforms single-modality baselines but also establishes a scalable paradigm for multi-modal medical image analysis, potentially reducing diagnostic variability and enhancing early intervention in laryngeal oncology.

Comparative analysis with prior studies underscores the advancements of our multi-modal fusion strategy in addressing the limitations of single- or dual-modality approaches. For instance, Li et al. (21). developed the Laryngopharyngeal Artificial Intelligence Diagnostic System (LPAIDS), a convolutional neural network-based model integrating white-light imaging (WLI) and NBI for real-time laryngeal and hypopharyngeal cancer detection, achieving accuracies of 95.6% in internal image tests and 94.0% in video-based evaluations, with external AUC-ROC values ranging from 0.965 to 0.987. While LPAIDS demonstrates excellent real-time performance and robustness across multi-center data, it primarily relies on raw image modalities without incorporating quantitative radiomics or clinical metadata, potentially limiting its ability to capture complementary contextual information. In contrast, our framework extends multi-modality to include 18 clinical features (e.g., tumor staging and smoking history) and 156 radiomic descriptors (e.g., GLCM contrast and gray-level run-length nonuniformity), fused via transformer encoders. This resulted in a 6.2 percentage point improvement over our best single-modality deep learning baseline (87.6% accuracy), highlighting the synergistic value of learned cross-attention over simple image fusion. Analytically, our model's attention allocation (47.0% to deep features for malignant cases) aligns with LPAIDS's emphasis on vascular patterns but enhances interpretability through modality-specific weighting, which could mitigate the subjective variability noted in their human-machine comparisons.

Similarly, Shuai et al. (2) proposed a deep learning fusion network combining structured medical records with laryngoscopic images for early glottic carcinoma diagnosis, achieving performance comparable to senior otolaryngologists and superior to monomodal models, with external validation across two centers. Their approach mirrors our integration of clinical data but omits radiomic quantification and advanced deep representations, relying instead on basic fusion techniques. Our study advances this by incorporating radiomics for texture and vascular heterogeneity analysis, features that contributed 34.7% attention in malignant classifications, and hierarchical deep embeddings from multiple architectures, yielding higher validation accuracy (93.8% vs. their reported human-level benchmarks). Ablation analyses in our work quantitatively demonstrate that excluding clinical features reduced accuracy by 1.7 percentage points (*p* = 0.002), while omitting radiomics or deep features led to larger drops (4.4% and 6.2%, respectively; both *p* < 0.001), emphasizing the analytical superiority of transformer-based fusion over their concatenation methods. This multi-faceted integration likely explains our framework's enhanced generalization (2.4% drop) compared to potential overfitting risks in record-image fusions, particularly in underserved settings where Shuai et al. (2). highlight utility.

In the domain of CE-NBI-specific classification, Esmaeili et al. (22) employed a fine-tuned ResNet50 model to categorize benign and malignant laryngeal lesions, achieving 83.5% testing accuracy on 8,181 images from 146 patients. Their focus on vascular pattern recognition via deep convolutional networks is conceptually aligned with our deep feature extraction, yet limited to single-modality analysis without feature selection or multi-modal enhancement. Our approach, utilizing CE-NBI as the imaging backbone, significantly outperforms this (93.8% vs. 83.5%) by fusing radiomics (e.g., GLCM metrics identified as top discriminators in SHAP analysis) and clinical data through six-layer transformers, reducing dimensionality via LASSO (retaining ∼35% of features) and improving specificity (94.6% vs. their implied lower metrics). Analytically, our five-fold cross-validation with external testing mitigates the data leakage risks in their cut-off-layer technique, while our model's smaller footprint post-fine-tuning (relative to full ResNet50) supports potential real-time deployment, extending their assisted detection paradigm.

Azam et al. (23) advanced real-time laryngeal squamous cell carcinoma detection using YOLOv5 on WL and NBI videolaryngoscopies, reporting a mean average precision of 0.63 and computation times of 0.026 s per frame, ideal for intraoperative use. While their ensemble model with test-time augmentation enhances detection speed and sensitivity, it lacks the depth of our multi-modal integration, focusing solely on bounding box localization without radiomic or clinical context. Our framework's higher accuracy (91.4% external) and sensitivity (90.0%) stem from cross-attention mechanisms that dynamically weight modalities, as evidenced by attention heatmaps showing emphasis on deep vascular features akin to their NBI enhancements. Comparatively, our ablation of transformer depth (optimal at 6 layers) vs. shallower alternatives (e.g., 2 layers: −3.6% accuracy, *p* < 0.001) analytically validates the need for complex inter-modal modeling, potentially complementing their video processing for hybrid systems.

Finally, Hu et al. (24) compared nasal endoscopy with optical biopsy (NBI, SPIES, iSCAN) and AI (EfficientNet B5) for early laryngeal cancer diagnosis, achieving 95.2% sensitivity and 96.5% specificity in a 142-patient cohort, reducing diagnostic delays from 15 to 7 days. Their retrospective AI application on vascular and textural features parallels our radiomic emphasis, but our study incorporates deeper multi-modal fusion, including clinical staging (ranked 6th in permutation importance) and hierarchical deep representations, yielding comparable specificity (92.5%) with broader generalizability across three centers. Analytically, our SHAP dependence plots reveal non-linear thresholds in deep features for malignancy prediction, extending their color-texture analysis and supporting reduced inter-rater variability (*κ*=0.84 in their study) through interpretable attention insights.

Collectively, these comparisons affirm our framework's novelty as the first to comprehensively fuse clinical, radiomic, and deep modalities via transformers for laryngeal lesion assessment, achieving state-of-the-art performance with enhanced interpretability and generalizability. Limitations include reliance on manual region-of-interest delineation and modest sample size, warranting prospective trials and expansion to multi-class subtyping. Future integrations with real-time video processing could further bridge gaps identified in prior works, ultimately advancing precision oncology in otolaryngology.

Several factors temper the interpretation of these results. Although the reported confidence intervals are relatively narrow, they were derived from patient-level bootstrapping and from metrics averaged across fifteen independent trials, which lowers estimate variance but does not fully compensate for the modest cohort size; the external estimates should therefore be viewed as encouraging rather than definitive. Overfitting was actively constrained through LASSO-based feature selection, dropout, early stopping, and strict patient-level data partitioning, and is further argued against by the small training-to-validation gap (2.3 percentage points) and the limited validation-to-external drop (2.4 percentage points). Larger multi-center cohorts and prospective validation nonetheless remain necessary to confirm these findings. In particular, the external cohort from Center C was isolated throughout model development and evaluated a single time after the final configuration was fixed, providing an unbiased generalization estimate that is not susceptible to the optimistic bias introduced by repeated evaluation on a tuned validation set. With respect to dimensionality, the 3,328 raw deep features are not classified directly but are first compressed through the transformer fusion module into a 256-dimensional joint representation, after which feature selection retains only 82 to 94 features; combined with the image-level training sample (6,047 internal images), this markedly reduces the effective feature-to-sample ratio and, together with LASSO regularization, mitigates the risk that the nominal feature dimensionality would otherwise pose.

From a translational standpoint, the framework is intended to function as a decision-support overlay rather than a standalone diagnostic tool, producing a calibrated malignancy probability alongside the live contact endoscopy examination while the clinician retains final interpretive control. Two boundaries of applicability should be emphasized. First, lesions smaller than 3 mm were excluded because adequate vascular pattern visualization could not be ensured, so performance on very small or very early lesions remains unverified. Second, the model relies on high-quality narrow band imaging with clearly resolved vascular detail, and frames degraded by motion artifact or bleeding were excluded during data collection; practical deployment would therefore benefit from an automated image-quality check that flags unsuitable frames before inference.

We also restricted the comparison to feature selection methods that preserve original feature identity; projection-based techniques such as principal component analysis and autoencoders could further reduce dimensionality but produce transformed or latent representations that obscure feature-level interpretation, and their use within an interpretable multi-modal pipeline represents a reasonable avenue for future investigation. Importantly, this study is retrospective and image-based; it does not include prospective validation, a real-time deployment experiment, a formal workflow-integration analysis, or a direct comparison against clinician performance. Claims regarding clinical applicability should therefore be interpreted as preliminary, and these evaluations represent essential prerequisites before the framework could be considered for clinical use.

## 5 Conclusion

This study establishes multi-modal transformer fusion as a new paradigm for laryngeal lesion classification, achieving 93.8% internal validation accuracy and 91.4% external test accuracy through systematic integration of clinical data, radiomic features, and deep learning representations. The framework significantly outperformed single-modality approaches, clinical features (71.3%), radiomics (84.2%), and deep learning alone (87.6%), with improvements of 22.5, 9.6, and 6.2 percentage points respectively. Superior generalization with only 2.4 percentage point validation-to-external drop compared to 5.7 percentage points for comparable studies demonstrates robustness gained through multi-modal integration. Comprehensive ablation studies confirmed each modality's unique contribution, with transformer fusion outperforming simple concatenation by 4.9 percentage points through learned cross-modal attention mechanisms. Feature importance analysis validated clinically established markers while demonstrating complementary value across modalities. Enhanced interpretability through explicit modality decomposition and attention visualization addresses critical barriers to clinical adoption of AI systems. From a clinical perspective, 90% sensitivity and 92.5% specificity with high positive (90.8%) and negative (92.3%) predictive values indicate strong utility for both identifying malignancies and confidently excluding cancer. Consistent multi-center performance suggests potential for use across diverse settings, although the present evaluation is retrospective and image-based, and actual clinical deployability would require dedicated validation. This work establishes methodological principles applicable beyond laryngeal diagnosis: multi-modal integration through transformer fusion for heterogeneous medical data, combination of handcrafted and learned features balancing interpretability and representational power, rigorous multi-center validation for credible clinical deployability assessment, and attention mechanisms enabling interpretable information weighting. Future extensions to prospective trials, multi-class subtyping, video analysis, and additional modalities will further advance computer-aided diagnosis in otolaryngology and establish multi-modal fusion as a generalizable paradigm for medical image analysis aimed at improving diagnostic accuracy and patient outcomes.

## Data Availability

The original contributions presented in the study are included in the article/Supplementary Material, further inquiries can be directed to the corresponding author.
