# Supplementary material for: A transformer-based multi-modal fusion framework for laryngeal lesion classification using contact endoscopy
Source: Front Surg. 2026 Sep 3;13:1840300. doi: 10.3389/fsurg.2026.1840300 (PMC13582240; doi:10.3389/fsurg.2026.1840300)
Supplement: Supplementary file 1 [file Table1.docx]

# ****Table S1.** Performance of Clinical Features Alone Across Feature Selection Methods and Classifiers**

| **Feature Selection** | **Classifier** | **Dataset** | **Acc (%)** | **Sens (%)** | **Spec (%)** | **F1 (%)** | **AUC** |
| --- | --- | --- | --- | --- | --- | --- | --- |
| **LASSO** | Vision Transformer | Training | 73.2 ± 2.1 | 70.1 ± 2.8 | 75.8 ± 2.5 | 71.4 ± 2.4 | 0.782 ± 0.024 |
|  |  | Validation | 70.8 ± 2.9 | 67.4 ± 3.5 | 73.6 ± 3.2 | 69.1 ± 3.1 | 0.751 ± 0.031 |
|  |  | External | 68.6 ± 3.2 | 64.7 ± 4.1 | 72.0 ± 3.8 | 67.2 ± 3.5 | 0.718 ± 0.037 |
|  | Attention-MLP | Training | 72.5 ± 2.3 | 69.3 ± 3.0 | 75.1 ± 2.7 | 70.7 ± 2.6 | 0.776 ± 0.026 |
|  |  | Validation | 70.2 ± 3.1 | 66.8 ± 3.6 | 73.1 ± 3.4 | 68.5 ± 3.3 | 0.745 ± 0.033 |
|  |  | External | 67.9 ± 3.4 | 64.0 ± 4.3 | 71.3 ± 4.0 | 66.4 ± 3.7 | 0.711 ± 0.039 |
|  | Graph Neural Net | Training | 71.3 ± 2.5 | 67.8 ± 3.2 | 74.2 ± 2.9 | 69.4 ± 2.8 | 0.763 ± 0.028 |
|  |  | Validation | 68.9 ± 3.3 | 65.2 ± 3.8 | 71.9 ± 3.5 | 67.1 ± 3.4 | 0.732 ± 0.035 |
|  |  | External | 66.4 ± 3.6 | 62.3 ± 4.5 | 69.8 ± 4.2 | 64.7 ± 3.9 | 0.701 ± 0.041 |
| **Mutual Info** | Vision Transformer | Training | 72.1 ± 2.2 | 68.7 ± 2.9 | 74.9 ± 2.6 | 70.5 ± 2.5 | 0.773 ± 0.025 |
|  |  | Validation | 69.6 ± 3.0 | 66.1 ± 3.6 | 72.5 ± 3.3 | 68.2 ± 3.2 | 0.742 ± 0.032 |
|  |  | External | 67.1 ± 3.5 | 63.3 ± 4.4 | 70.5 ± 4.1 | 65.8 ± 3.8 | 0.708 ± 0.040 |
|  | Attention-MLP | Training | 72.3 ± 2.3 | 69.0 ± 3.0 | 75.0 ± 2.7 | 70.6 ± 2.6 | 0.775 ± 0.026 |
|  |  | Validation | 69.8 ± 3.2 | 66.5 ± 3.7 | 72.7 ± 3.4 | 68.4 ± 3.3 | 0.744 ± 0.034 |
|  |  | External | 67.4 ± 3.5 | 63.7 ± 4.4 | 70.8 ± 4.1 | 66.1 ± 3.8 | 0.710 ± 0.040 |
|  | Graph Neural Net | Training | 71.8 ± 2.4 | 68.4 ± 3.1 | 74.6 ± 2.8 | 69.9 ± 2.7 | 0.770 ± 0.027 |
|  |  | Validation | 69.4 ± 3.2 | 65.9 ± 3.7 | 72.3 ± 3.5 | 67.9 ± 3.4 | 0.739 ± 0.034 |
|  |  | External | 66.9 ± 3.6 | 63.0 ± 4.5 | 70.3 ± 4.2 | 65.5 ± 3.9 | 0.705 ± 0.041 |
| **ReliefF** | Vision Transformer | Training | 73.6 ± 2.0 | 70.7 ± 2.7 | 76.0 ± 2.4 | 71.9 ± 2.3 | 0.785 ± 0.023 |
|  |  | Validation | 71.1 ± 2.8 | 68.2 ± 3.4 | 73.5 ± 3.0 | 69.6 ± 3.0 | 0.753 ± 0.030 |
|  |  | External | 68.3 ± 3.3 | 64.3 ± 4.2 | 71.8 ± 3.9 | 66.9 ± 3.6 | 0.720 ± 0.038 |
|  | **Attention-MLP** | Training | **73.9 ± 2.0** | **71.2 ± 2.7** | **76.1 ± 2.4** | **72.4 ± 2.3** | **0.788 ± 0.023** |
|  |  | Validation | **71.3 ± 2.8** | **68.9 ± 3.4** | **73.2 ± 3.1** | **70.1 ± 3.0** | **0.756 ± 0.029** |
|  |  | External | **68.7 ± 3.2** | **65.3 ± 4.1** | **71.8 ± 3.8** | **67.8 ± 3.5** | **0.723 ± 0.037** |
|  | Graph Neural Net | Training | 72.9 ± 2.2 | 69.8 ± 2.9 | 75.4 ± 2.6 | 71.1 ± 2.5 | 0.779 ± 0.025 |
|  |  | Validation | 70.4 ± 3.0 | 67.3 ± 3.5 | 72.9 ± 3.2 | 69.2 ± 3.1 | 0.748 ± 0.031 |
|  |  | External | 67.7 ± 3.4 | 63.7 ± 4.3 | 71.3 ± 4.0 | 66.3 ± 3.7 | 0.715 ± 0.039 |

Values are mean ± standard deviation across 5 folds × 3 repetitions (15 runs total). Best results in bold. Training set performance evaluated on 80% of internal data per fold. Acc: Accuracy, Sens: Sensitivity, Spec: Specificity, F1: F1-score, AUC: Area under ROC curve. Number of features selected: LASSO=12, Mutual Info=10, ReliefF=11.

# ****Table S2.** Performance of Radiomic Features Alone Across Feature Selection Methods and Classifiers**

| **Feature Selection** | **Classifier** | **Dataset** | **Acc (%)** | **Sens (%)** | **Spec (%)** | **F1 (%)** | **AUC** |
| --- | --- | --- | --- | --- | --- | --- | --- |
| **LASSO** | Vision Transformer | Training | 87.3 ± 1.5 | 85.9 ± 2.1 | 88.4 ± 1.8 | 86.5 ± 1.9 | 0.931 ± 0.013 |
|  |  | Validation | 84.2 ± 2.1 | 82.6 ± 2.7 | 85.5 ± 2.3 | 83.4 ± 2.4 | 0.901 ± 0.018 |
|  |  | External | 81.4 ± 2.8 | 79.2 ± 3.6 | 83.1 ± 3.2 | 80.3 ± 3.1 | 0.882 ± 0.031 |
|  | Attention-MLP | Training | 86.8 ± 1.6 | 85.3 ± 2.2 | 88.0 ± 1.9 | 86.0 ± 2.0 | 0.927 ± 0.014 |
|  |  | Validation | 83.8 ± 2.2 | 82.1 ± 2.8 | 85.2 ± 2.4 | 82.9 ± 2.5 | 0.897 ± 0.019 |
|  |  | External | 80.9 ± 2.9 | 78.6 ± 3.7 | 82.8 ± 3.3 | 79.7 ± 3.2 | 0.877 ± 0.032 |
|  | Graph Neural Net | Training | 85.7 ± 1.8 | 84.1 ± 2.4 | 86.9 ± 2.1 | 84.9 ± 2.2 | 0.918 ± 0.016 |
|  |  | Validation | 82.9 ± 2.4 | 81.3 ± 2.9 | 84.2 ± 2.6 | 82.1 ± 2.6 | 0.889 ± 0.021 |
|  |  | External | 79.7 ± 3.1 | 77.3 ± 3.9 | 81.8 ± 3.5 | 78.5 ± 3.4 | 0.868 ± 0.034 |
| **Mutual Info** | Vision Transformer | Training | 86.9 ± 1.7 | 85.5 ± 2.2 | 88.1 ± 2.0 | 86.2 ± 2.0 | 0.928 ± 0.015 |
|  |  | Validation | 83.9 ± 2.3 | 82.4 ± 2.8 | 85.1 ± 2.5 | 83.1 ± 2.5 | 0.898 ± 0.020 |
|  |  | External | 81.1 ± 2.9 | 78.9 ± 3.7 | 82.9 ± 3.3 | 79.9 ± 3.2 | 0.879 ± 0.032 |
|  | Attention-MLP | Training | 86.6 ± 1.7 | 85.1 ± 2.3 | 87.8 ± 2.0 | 85.9 ± 2.1 | 0.925 ± 0.015 |
|  |  | Validation | 83.7 ± 2.3 | 82.0 ± 2.9 | 85.0 ± 2.5 | 82.8 ± 2.6 | 0.895 ± 0.020 |
|  |  | External | 80.7 ± 3.0 | 78.3 ± 3.8 | 82.6 ± 3.4 | 79.4 ± 3.3 | 0.875 ± 0.033 |
|  | Graph Neural Net | Training | 85.9 ± 1.9 | 84.3 ± 2.5 | 87.2 ± 2.2 | 85.1 ± 2.3 | 0.920 ± 0.017 |
|  |  | Validation | 83.1 ± 2.5 | 81.5 ± 3.0 | 84.4 ± 2.7 | 82.3 ± 2.7 | 0.891 ± 0.022 |
|  |  | External | 80.0 ± 3.1 | 77.7 ± 3.9 | 82.0 ± 3.5 | 78.8 ± 3.4 | 0.871 ± 0.034 |
| **ReliefF** | Vision Transformer | Training | 86.3 ± 1.8 | 84.7 ± 2.4 | 87.6 ± 2.1 | 85.5 ± 2.2 | 0.923 ± 0.016 |
|  |  | Validation | 83.4 ± 2.4 | 81.8 ± 2.9 | 84.7 ± 2.6 | 82.6 ± 2.6 | 0.893 ± 0.021 |
|  |  | External | 80.4 ± 3.0 | 78.1 ± 3.8 | 82.3 ± 3.4 | 79.2 ± 3.3 | 0.873 ± 0.033 |
|  | Attention-MLP | Training | 86.0 ± 1.8 | 84.3 ± 2.4 | 87.4 ± 2.1 | 85.1 ± 2.2 | 0.921 ± 0.016 |
|  |  | Validation | 83.2 ± 2.4 | 81.5 ± 2.9 | 84.5 ± 2.6 | 82.3 ± 2.7 | 0.891 ± 0.021 |
|  |  | External | 80.0 ± 3.1 | 77.5 ± 3.9 | 82.1 ± 3.5 | 78.7 ± 3.4 | 0.870 ± 0.034 |
|  | Graph Neural Net | Training | 85.7 ± 1.9 | 84.0 ± 2.5 | 87.0 ± 2.2 | 84.8 ± 2.3 | 0.918 ± 0.017 |
|  |  | Validation | 82.9 ± 2.4 | 81.2 ± 3.0 | 84.3 ± 2.6 | 82.0 ± 2.7 | 0.888 ± 0.022 |
|  |  | External | 79.6 ± 3.1 | 77.0 ± 3.9 | 81.7 ± 3.5 | 78.3 ± 3.4 | 0.866 ± 0.035 |

Values are mean ± standard deviation across 5 folds × 3 repetitions. Best results in bold. Number of features selected: LASSO=47, Mutual Info=52, ReliefF=44.

# ****Table S3.** Performance of Individual ConvNeXt V2 Features Across Feature Selection Methods and Classifiers**

| **Feature Selection** | **Classifier** | **Dataset** | **Acc (%)** | **Sens (%)** | **Spec (%)** | **F1 (%)** | **AUC** |
| --- | --- | --- | --- | --- | --- | --- | --- |
| **LASSO** | Vision Transformer | Training | 90.2 ± 1.4 | 88.9 ± 1.9 | 91.2 ± 1.6 | 89.5 ± 1.7 | 0.964 ± 0.011 |
|  |  | Validation | 87.6 ± 1.9 | 86.2 ± 2.4 | 88.7 ± 2.1 | 86.9 ± 2.2 | 0.941 ± 0.016 |
|  |  | External | 85.7 ± 2.5 | 84.2 ± 3.2 | 86.9 ± 2.9 | 85.0 ± 2.8 | 0.921 ± 0.027 |
|  | Attention-MLP | Training | 89.7 ± 1.5 | 88.3 ± 2.0 | 90.8 ± 1.7 | 89.0 ± 1.8 | 0.960 ± 0.012 |
|  |  | Validation | 87.2 ± 2.0 | 85.7 ± 2.5 | 88.4 ± 2.2 | 86.4 ± 2.3 | 0.937 ± 0.017 |
|  |  | External | 85.1 ± 2.6 | 83.5 ± 3.3 | 86.4 ± 3.0 | 84.3 ± 2.9 | 0.916 ± 0.028 |
|  | Graph Neural Net | Training | 88.9 ± 1.6 | 87.4 ± 2.1 | 90.1 ± 1.8 | 88.2 ± 1.9 | 0.953 ± 0.013 |
|  |  | Validation | 86.5 ± 2.1 | 84.9 ± 2.6 | 87.8 ± 2.3 | 85.7 ± 2.4 | 0.930 ± 0.018 |
|  |  | External | 84.3 ± 2.7 | 82.6 ± 3.4 | 85.7 ± 3.1 | 83.5 ± 3.0 | 0.908 ± 0.029 |
| **Mutual Info** | Vision Transformer | Training | 89.8 ± 1.5 | 88.5 ± 2.0 | 90.9 ± 1.7 | 89.1 ± 1.8 | 0.961 ± 0.012 |
|  |  | Validation | 87.3 ± 2.0 | 85.9 ± 2.5 | 88.5 ± 2.2 | 86.6 ± 2.3 | 0.938 ± 0.017 |
|  |  | External | 85.4 ± 2.6 | 83.8 ± 3.3 | 86.6 ± 3.0 | 84.6 ± 2.9 | 0.918 ± 0.028 |
|  | Attention-MLP | Training | 89.6 ± 1.5 | 88.1 ± 2.0 | 90.7 ± 1.7 | 88.9 ± 1.8 | 0.959 ± 0.012 |
|  |  | Validation | 87.1 ± 2.0 | 85.6 ± 2.5 | 88.3 ± 2.2 | 86.3 ± 2.3 | 0.936 ± 0.017 |
|  |  | External | 85.0 ± 2.6 | 83.3 ± 3.3 | 86.3 ± 3.0 | 84.2 ± 2.9 | 0.915 ± 0.028 |
|  | Graph Neural Net | Training | 89.1 ± 1.6 | 87.6 ± 2.1 | 90.3 ± 1.8 | 88.4 ± 1.9 | 0.955 ± 0.013 |
|  |  | Validation | 86.7 ± 2.1 | 85.1 ± 2.6 | 88.0 ± 2.3 | 85.9 ± 2.4 | 0.932 ± 0.018 |
|  |  | External | 84.6 ± 2.7 | 82.9 ± 3.4 | 85.9 ± 3.1 | 83.8 ± 3.0 | 0.911 ± 0.029 |
| **ReliefF** | Vision Transformer | Training | 89.7 ± 1.5 | 88.3 ± 2.0 | 90.8 ± 1.7 | 89.0 ± 1.8 | 0.960 ± 0.012 |
|  |  | Validation | 87.2 ± 2.0 | 85.8 ± 2.5 | 88.4 ± 2.2 | 86.5 ± 2.3 | 0.937 ± 0.017 |
|  |  | External | 85.3 ± 2.6 | 83.7 ± 3.3 | 86.5 ± 3.0 | 84.5 ± 2.9 | 0.917 ± 0.028 |
|  | Attention-MLP | Training | 89.4 ± 1.6 | 87.9 ± 2.1 | 90.6 ± 1.8 | 88.7 ± 1.9 | 0.957 ± 0.013 |
|  |  | Validation | 86.9 ± 2.1 | 85.4 ± 2.6 | 88.1 ± 2.3 | 86.1 ± 2.4 | 0.934 ± 0.018 |
|  |  | External | 84.9 ± 2.7 | 83.1 ± 3.4 | 86.2 ± 3.1 | 84.0 ± 3.0 | 0.913 ± 0.029 |
|  | Graph Neural Net | Training | 89.2 ± 1.6 | 87.7 ± 2.1 | 90.4 ± 1.8 | 88.5 ± 1.9 | 0.956 ± 0.013 |
|  |  | Validation | 86.8 ± 2.1 | 85.2 ± 2.6 | 88.0 ± 2.3 | 86.0 ± 2.4 | 0.933 ± 0.018 |
|  |  | External | 84.7 ± 2.7 | 82.8 ± 3.4 | 86.0 ± 3.1 | 83.8 ± 3.0 | 0.911 ± 0.029 |

# ****Table S4.** Performance of Individual Swin Transformer V2 Features Across Feature Selection Methods and Classifiers Feature Selection**

| **Feature Selection** | **Classifier** | **Dataset** | **Acc (%)** | **Sens (%)** | **Spec (%)** | **F1 (%)** | **AUC** |
| --- | --- | --- | --- | --- | --- | --- | --- |
| **LASSO** | Vision Transformer | Training | 89.0 ± 1.6 | 87.5 ± 2.1 | 90.2 ± 1.8 | 88.3 ± 1.9 | 0.953 ± 0.013 |
|  |  | Validation | 86.4 ± 2.1 | 84.9 ± 2.6 | 87.6 ± 2.3 | 85.6 ± 2.4 | 0.930 ± 0.018 |
|  |  | External | 84.3 ± 2.7 | 82.6 ± 3.4 | 85.7 ± 3.1 | 83.5 ± 3.0 | 0.908 ± 0.029 |
|  | Attention-MLP | Training | 88.7 ± 1.6 | 87.1 ± 2.1 | 89.9 ± 1.8 | 87.9 ± 1.9 | 0.950 ± 0.014 |
|  |  | Validation | 86.2 ± 2.1 | 84.6 ± 2.6 | 87.4 ± 2.3 | 85.4 ± 2.4 | 0.927 ± 0.019 |
|  |  | External | 83.9 ± 2.7 | 82.1 ± 3.4 | 85.3 ± 3.1 | 83.0 ± 3.0 | 0.904 ± 0.030 |
|  | Graph Neural Net | Training | 88.1 ± 1.7 | 86.5 ± 2.2 | 89.4 ± 1.9 | 87.3 ± 2.0 | 0.944 ± 0.015 |
|  |  | Validation | 85.7 ± 2.2 | 84.0 ± 2.7 | 87.0 ± 2.4 | 84.8 ± 2.5 | 0.921 ± 0.020 |
|  |  | External | 83.1 ± 2.8 | 81.2 ± 3.5 | 84.6 ± 3.2 | 82.2 ± 3.1 | 0.897 ± 0.031 |
| **Mutual Info** | Vision Transformer | Training | 89.1 ± 1.6 | 87.6 ± 2.1 | 90.3 ± 1.8 | 88.4 ± 1.9 | 0.954 ± 0.013 |
|  |  | Validation | 86.5 ± 2.1 | 85.0 ± 2.6 | 87.7 ± 2.3 | 85.7 ± 2.4 | 0.931 ± 0.018 |
|  |  | External | 84.4 ± 2.7 | 82.7 ± 3.4 | 85.8 ± 3.1 | 83.6 ± 3.0 | 0.909 ± 0.029 |
|  | Attention-MLP | Training | 88.8 ± 1.6 | 87.2 ± 2.1 | 90.0 ± 1.8 | 88.0 ± 1.9 | 0.952 ± 0.014 |
|  |  | Validation | 86.3 ± 2.1 | 84.7 ± 2.6 | 87.5 ± 2.3 | 85.5 ± 2.4 | 0.929 ± 0.019 |
|  |  | External | 84.1 ± 2.7 | 82.3 ± 3.4 | 85.5 ± 3.1 | 83.2 ± 3.0 | 0.906 ± 0.030 |
|  | Graph Neural Net | Training | 88.6 ± 1.7 | 87.0 ± 2.2 | 89.8 ± 1.9 | 87.8 ± 2.0 | 0.950 ± 0.014 |
|  |  | Validation | 86.2 ± 2.2 | 84.5 ± 2.7 | 87.4 ± 2.4 | 85.3 ± 2.5 | 0.927 ± 0.019 |
|  |  | External | 83.8 ± 2.8 | 81.9 ± 3.5 | 85.2 ± 3.2 | 82.9 ± 3.1 | 0.903 ± 0.031 |
| **ReliefF** | Vision Transformer | Training | 89.2 ± 1.6 | 87.8 ± 2.1 | 90.4 ± 1.8 | 88.5 ± 1.9 | 0.956 ± 0.013 |
|  |  | Validation | 86.7 ± 2.0 | 85.2 ± 2.5 | 87.9 ± 2.2 | 85.9 ± 2.3 | 0.933 ± 0.018 |
|  |  | External | 84.5 ± 2.6 | 82.9 ± 3.3 | 85.9 ± 3.0 | 83.7 ± 2.9 | 0.910 ± 0.029 |
|  | Attention-MLP | Training | 89.0 ± 1.6 | 87.5 ± 2.1 | 90.2 ± 1.8 | 88.3 ± 1.9 | 0.954 ± 0.013 |
|  |  | Validation | 86.9 ± 2.0 | 85.5 ± 2.5 | 88.0 ± 2.2 | 86.2 ± 2.3 | 0.934 ± 0.017 |
|  |  | External | 84.3 ± 2.6 | 82.8 ± 3.3 | 85.5 ± 3.0 | 83.6 ± 2.9 | 0.908 ± 0.028 |
|  | Graph Neural Net | Training | 88.8 ± 1.6 | 87.3 ± 2.1 | 90.0 ± 1.8 | 88.1 ± 1.9 | 0.953 ± 0.013 |
|  |  | Validation | 86.4 ± 2.1 | 84.9 ± 2.6 | 87.6 ± 2.3 | 85.6 ± 2.4 | 0.930 ± 0.018 |
|  |  | External | 84.0 ± 2.7 | 82.3 ± 3.4 | 85.3 ± 3.1 | 83.2 ± 3.0 | 0.905 ± 0.029 |

# ****Table S5.** Performance of Individual EfficientNet V2 Features Across Feature Selection Methods and Classifiers Feature Selection**

| **Feature Selection** | **Classifier** | **Dataset** | **Acc (%)** | **Sens (%)** | **Spec (%)** | **F1 (%)** | **AUC** |
| --- | --- | --- | --- | --- | --- | --- | --- |
| **LASSO** | Vision Transformer | Training | 87.8 ± 1.7 | 86.2 ± 2.3 | 89.1 ± 2.0 | 87.0 ± 2.1 | 0.941 ± 0.015 |
|  |  | Validation | 85.3 ± 2.4 | 83.6 ± 2.9 | 86.7 ± 2.6 | 84.5 ± 2.7 | 0.918 ± 0.021 |
|  |  | External | 83.1 ± 2.9 | 81.2 ± 3.7 | 84.6 ± 3.3 | 82.2 ± 3.2 | 0.897 ± 0.032 |
|  | Attention-MLP | Training | 87.4 ± 1.8 | 85.7 ± 2.3 | 88.8 ± 2.0 | 86.6 ± 2.1 | 0.938 ± 0.015 |
|  |  | Validation | 85.0 ± 2.4 | 83.2 ± 2.9 | 86.5 ± 2.6 | 84.1 ± 2.7 | 0.915 ± 0.021 |
|  |  | External | 82.7 ± 3.0 | 80.7 ± 3.7 | 84.3 ± 3.4 | 81.7 ± 3.2 | 0.893 ± 0.033 |
|  | Graph Neural Net | Training | 86.9 ± 1.9 | 85.1 ± 2.4 | 88.3 ± 2.1 | 86.1 ± 2.2 | 0.932 ± 0.016 |
|  |  | Validation | 84.5 ± 2.5 | 82.6 ± 3.0 | 86.0 ± 2.7 | 83.6 ± 2.8 | 0.909 ± 0.022 |
|  |  | External | 81.9 ± 3.1 | 79.8 ± 3.8 | 83.6 ± 3.5 | 80.8 ± 3.3 | 0.885 ± 0.034 |
| **Mutual Info** | Vision Transformer | Training | 88.1 ± 1.7 | 86.5 ± 2.3 | 89.4 ± 2.0 | 87.3 ± 2.1 | 0.944 ± 0.015 |
|  |  | Validation | 85.6 ± 2.3 | 84.0 ± 2.8 | 86.9 ± 2.5 | 84.8 ± 2.6 | 0.921 ± 0.020 |
|  |  | External | 83.4 ± 2.9 | 81.6 ± 3.6 | 84.9 ± 3.3 | 82.5 ± 3.1 | 0.900 ± 0.032 |
|  | Attention-MLP | Training | 88.2 ± 1.7 | 86.7 ± 2.3 | 89.5 ± 2.0 | 87.5 ± 2.1 | 0.946 ± 0.015 |
|  |  | Validation | 85.7 ± 2.3 | 84.2 ± 2.8 | 87.0 ± 2.5 | 85.0 ± 2.6 | 0.923 ± 0.020 |
|  |  | External | 83.1 ± 2.9 | 81.4 ± 3.6 | 84.6 ± 3.3 | 82.3 ± 3.1 | 0.897 ± 0.032 |
|  | Graph Neural Net | Training | 87.7 ± 1.8 | 86.0 ± 2.4 | 89.0 ± 2.1 | 86.9 ± 2.2 | 0.940 ± 0.016 |
|  |  | Validation | 85.2 ± 2.4 | 83.5 ± 2.9 | 86.6 ± 2.6 | 84.4 ± 2.7 | 0.917 ± 0.021 |
|  |  | External | 82.7 ± 3.0 | 80.9 ± 3.7 | 84.2 ± 3.4 | 81.8 ± 3.2 | 0.893 ± 0.033 |
| **ReliefF** | Vision Transformer | Training | 87.6 ± 1.8 | 86.0 ± 2.3 | 88.9 ± 2.0 | 86.8 ± 2.1 | 0.939 ± 0.016 |
|  |  | Validation | 85.1 ± 2.4 | 83.4 ± 2.9 | 86.6 ± 2.6 | 84.3 ± 2.7 | 0.916 ± 0.021 |
|  |  | External | 82.9 ± 3.0 | 81.0 ± 3.7 | 84.4 ± 3.4 | 82.0 ± 3.2 | 0.895 ± 0.033 |
|  | Attention-MLP | Training | 87.8 ± 1.8 | 86.2 ± 2.3 | 89.1 ± 2.0 | 87.0 ± 2.1 | 0.942 ± 0.015 |
|  |  | Validation | 85.3 ± 2.4 | 83.7 ± 2.9 | 86.8 ± 2.6 | 84.6 ± 2.7 | 0.919 ± 0.021 |
|  |  | External | 83.0 ± 2.9 | 81.2 ± 3.6 | 84.5 ± 3.3 | 82.1 ± 3.1 | 0.896 ± 0.032 |
|  | Graph Neural Net | Training | 87.3 ± 1.9 | 85.6 ± 2.4 | 88.6 ± 2.1 | 86.5 ± 2.2 | 0.937 ± 0.016 |
|  |  | Validation | 84.9 ± 2.5 | 83.1 ± 3.0 | 86.4 ± 2.7 | 84.0 ± 2.8 | 0.914 ± 0.022 |
|  |  | External | 82.4 ± 3.1 | 80.4 ± 3.8 | 83.9 ± 3.5 | 81.4 ± 3.3 | 0.890 ± 0.034 |

# ****Table S6.**** **Performance of Multi-Modal Transformer Fusion Across Feature Selection Methods and Classifiers**

| **Feature Selection** | **Classifier** | **Dataset** | **Acc (%)** | **Sens (%)** | **Spec (%)** | **Prec (%)** | **F1 (%)** | **MCC** | **AUC** |
| --- | --- | --- | --- | --- | --- | --- | --- | --- | --- |
| **LASSO** | Vision Transformer | Training | 96.1 ± 1.0 | 95.2 ± 1.4 | 96.8 ± 1.2 | 96.3 ± 1.1 | 95.7 ± 1.2 | 0.921 ± 0.021 | 0.992 ± 0.007 |
|  |  | Validation | 93.8 ± 1.4 | 92.7 ± 1.8 | 94.6 ± 1.6 | 93.9 ± 1.5 | 93.3 ± 1.6 | 0.874 ± 0.029 | 0.981 ± 0.012 |
|  |  | External | 91.4 ± 2.1 | 90.0 ± 2.8 | 92.5 ± 2.5 | 90.8 ± 2.4 | 90.4 ± 2.6 | 0.825 ± 0.043 | 0.967 ± 0.019 |
|  | Attention-MLP | Training | 95.7 ± 1.1 | 94.6 ± 1.5 | 96.5 ± 1.3 | 95.9 ± 1.2 | 95.2 ± 1.3 | 0.913 ± 0.023 | 0.989 ± 0.008 |
|  |  | Validation | 93.4 ± 1.5 | 92.1 ± 1.9 | 94.4 ± 1.7 | 93.5 ± 1.6 | 92.8 ± 1.7 | 0.866 ± 0.031 | 0.978 ± 0.013 |
|  |  | External | 90.9 ± 2.2 | 89.3 ± 2.9 | 92.1 ± 2.6 | 90.2 ± 2.5 | 89.7 ± 2.7 | 0.817 ± 0.045 | 0.963 ± 0.020 |
|  | Graph Neural Net | Training | 95.0 ± 1.2 | 93.7 ± 1.7 | 95.9 ± 1.4 | 95.1 ± 1.3 | 94.4 ± 1.5 | 0.899 ± 0.025 | 0.984 ± 0.009 |
|  |  | Validation | 92.7 ± 1.6 | 91.3 ± 2.0 | 93.8 ± 1.8 | 92.7 ± 1.7 | 92.0 ± 1.8 | 0.852 ± 0.033 | 0.972 ± 0.014 |
|  |  | External | 90.0 ± 2.4 | 88.3 ± 3.1 | 91.3 ± 2.8 | 89.2 ± 2.7 | 88.7 ± 2.9 | 0.799 ± 0.048 | 0.956 ± 0.022 |
| **Mutual Info** | Vision Transformer | Training | 95.8 ± 1.1 | 94.8 ± 1.5 | 96.5 ± 1.3 | 96.0 ± 1.2 | 95.4 ± 1.3 | 0.915 ± 0.022 | 0.990 ± 0.008 |
|  |  | Validation | 93.5 ± 1.5 | 92.3 ± 1.9 | 94.4 ± 1.7 | 93.6 ± 1.6 | 92.9 ± 1.7 | 0.868 ± 0.030 | 0.979 ± 0.013 |
|  |  | External | 91.1 ± 2.2 | 89.7 ± 2.9 | 92.3 ± 2.6 | 90.5 ± 2.5 | 90.1 ± 2.7 | 0.821 ± 0.044 | 0.965 ± 0.020 |
|  | Attention-MLP | Training | 95.5 ± 1.1 | 94.4 ± 1.6 | 96.3 ± 1.3 | 95.7 ± 1.3 | 95.0 ± 1.4 | 0.909 ± 0.023 | 0.988 ± 0.009 |
|  |  | Validation | 93.2 ± 1.5 | 91.9 ± 1.9 | 94.2 ± 1.7 | 93.2 ± 1.6 | 92.5 ± 1.7 | 0.862 ± 0.031 | 0.976 ± 0.013 |
|  |  | External | 90.7 ± 2.3 | 89.0 ± 3.0 | 92.0 ± 2.7 | 89.9 ± 2.6 | 89.4 ± 2.8 | 0.813 ± 0.046 | 0.961 ± 0.021 |
|  | Graph Neural Net | Training | 94.7 ± 1.3 | 93.4 ± 1.8 | 95.7 ± 1.5 | 94.8 ± 1.4 | 94.1 ± 1.6 | 0.893 ± 0.027 | 0.982 ± 0.010 |
|  |  | Validation | 92.4 ± 1.7 | 91.0 ± 2.1 | 93.5 ± 1.8 | 92.4 ± 1.8 | 91.7 ± 1.9 | 0.847 ± 0.034 | 0.970 ± 0.015 |
|  |  | External | 89.6 ± 2.5 | 87.7 ± 3.2 | 91.0 ± 2.9 | 88.6 ± 2.8 | 88.1 ± 3.0 | 0.791 ± 0.050 | 0.953 ± 0.023 |
| **ReliefF** | Vision Transformer | Training | 95.2 ± 1.2 | 94.1 ± 1.6 | 96.0 ± 1.4 | 95.4 ± 1.3 | 94.7 ± 1.4 | 0.903 ± 0.024 | 0.986 ± 0.009 |
|  |  | Validation | 92.9 ± 1.6 | 91.6 ± 2.0 | 94.0 ± 1.8 | 93.0 ± 1.7 | 92.3 ± 1.8 | 0.857 ± 0.032 | 0.974 ± 0.014 |
|  |  | External | 90.4 ± 2.3 | 88.7 ± 3.0 | 91.8 ± 2.7 | 89.7 ± 2.6 | 89.2 ± 2.8 | 0.807 ± 0.046 | 0.959 ± 0.021 |
|  | Attention-MLP | Training | 94.9 ± 1.3 | 93.6 ± 1.7 | 95.8 ± 1.5 | 95.0 ± 1.4 | 94.3 ± 1.5 | 0.897 ± 0.026 | 0.983 ± 0.010 |
|  |  | Validation | 92.6 ± 1.7 | 91.2 ± 2.1 | 93.7 ± 1.8 | 92.6 ± 1.8 | 91.9 ± 1.9 | 0.851 ± 0.033 | 0.971 ± 0.015 |
|  |  | External | 89.9 ± 2.4 | 88.0 ± 3.1 | 91.4 ± 2.8 | 89.1 ± 2.7 | 88.5 ± 2.9 | 0.798 ± 0.049 | 0.955 ± 0.022 |
|  | Graph Neural Net | Training | 93.7 ± 1.5 | 92.2 ± 2.0 | 94.9 ± 1.7 | 93.6 ± 1.6 | 92.9 ± 1.8 | 0.873 ± 0.031 | 0.976 ± 0.012 |
|  |  | Validation | 91.4 ± 1.9 | 89.8 ± 2.3 | 92.6 ± 2.1 | 91.3 ± 2.0 | 90.5 ± 2.1 | 0.826 ± 0.038 | 0.963 ± 0.017 |
|  |  | External | 88.6 ± 2.6 | 86.3 ± 3.4 | 90.3 ± 3.0 | 87.4 ± 2.9 | 86.8 ± 3.1 | 0.771 ± 0.053 | 0.947 ± 0.024 |

Values are mean ± standard deviation for internal validation across 5 folds × 3 repetitions. Best results in bold. Acc: Accuracy, Sens: Sensitivity, Spec: Specificity, Prec: Precision, F1: F1-score, MCC: Matthews correlation coefficient, AUC: Area under ROC curve. Number of features selected: LASSO=89, Mutual Info=94, ReliefF=82.

# ****Figure S1.** Performance of Swin Transformer V2 Features Using Different Feature Selection and Classifiers**

# ****Figure S2.** Performance of EfficientNet V2 Features Using Different Feature Selection and Classifiers**
